# Underweight Status Amplifies Respiratory Infection Mortality in Diabetes: Findings From a Nationwide Cohort Study

**DOI:** 10.1002/jcsm.70263

**Published:** 2026-03-28

**Authors:** Hun Jee Choe, Kyong Do Han, Ji‐Hong Park, Jiwoo Lee, Mi Kyung Kwak, Yun Mi Choi, Sun‐Joon Moon, Eun‐Gyoung Hong

**Affiliations:** ^1^ Division of Endocrinology and Metabolism, Department of Internal Medicine Hallym University Dongtan Sacred Heart Hospital Hwaseong‐si Gyeonggi‐do South Korea; ^2^ Department of Statistics and Actuarial Science Soongsil University Seoul Dongjak‐gu Republic of Korea; ^3^ Division of Endocrinology and Metabolism, Department of Internal Medicine, Kangbuk Samsung Hospital, Sungkyunkwan University School of Medicine Seoul Republic of Korea

**Keywords:** COVID‐19, diabetes, mortality, respiratory infection, tuberculosis, underweight

## Abstract

**Background:**

Diabetes increases susceptibility to infectious respiratory diseases, but the impact of underweight status on mortality from these conditions remains unclear. We evaluated the association between low body mass index (BMI) and respiratory infection‐related mortality in a large nationwide cohort of individuals with diabetes.

**Methods:**

We conducted a population‐based cohort study using linked Korean national health and mortality data, including 2 508 409 adults with type 2 diabetes. Participants were stratified by BMI and followed for cause‐specific mortality from influenza/pneumonia, tuberculosis and COVID‐19. Cox proportional hazards models were used to estimate adjusted hazard ratios (aHRs), accounting for demographics, lifestyle factors, comorbidities and competing risks.

**Results:**

Over a median follow‐up of 6 years, 18 024 (0.72%) participants died due to respiratory infections. Compared to individuals with BMI ≥ 18.5 kg/m^2^, the aHRs for respiratory mortality were 7.27 (95% CI, 6.31–8.38), 4.79 (4.25–5.41) and 3.15 (2.93–3.38) for BMI < 16, 16–16.9 and 17–18.4 kg/m^2^, respectively (all *p* < 0.001) with a progressive risk gradient. Underweight status amplified the risk of tuberculosis‐related mortality most markedly (aHR, 9.93 for BMI < 16 kg/m^2^, *p* < 0.001). Mortality risks in underweight individuals exceeded those for BMI ≥ 35 kg/m^2^ relative to the reference group (25–29.9 kg/m^2^). Associations were more pronounced in individuals under 65 years and those without cardiovascular or kidney disease.

**Conclusions:**

Underweight status is a strong, independent predictor of respiratory infection‐related mortality in individuals with diabetes. Recognition of low BMI as a marker of vulnerability may improve targeted preventive strategies in diabetes care.

**Abbreviations**
aHR: adjusted hazard ratio
ALT: alanine aminotransferase
AST: aspartate aminotransferase
BMI: body mass index
CI: confidence interval
CKD: chronic kidney disease
COPD: chronic obstructive pulmonary disease
COVID‐19: coronavirus disease 2019
CVD: cardiovascular disease
DBP: diastolic blood pressure
eGFR: estimated glomerular filtration rate
HDL: high‐density lipoprotein cholesterol
ICD‐10: International Classification of Diseases, 10th Revision
IR: incidence rate
LDL: low‐density lipoprotein cholesterol
NHIS: National Health Insurance Service
SBP: systolic blood pressure
SD: standard deviation
T2DM: type 2 diabetes mellitus
TB: tuberculosis

## 1 Introduction

Underweight status is increasingly recognized as a critical risk factor for infection‐related morbidity and mortality, particularly among vulnerable populations [1, 2]. Although obesity is widely recognized for its association with various health risks, the relationship between body weight and overall mortality remains complex [3, 4]. In a large‐scale cohort study of over 1.3 million adults, both underweight and overweight individuals exhibited higher all‐cause mortality rates compared to those with normal weight [5]. While cancer and cardiovascular diseases (CVDs) were the primary contributors to mortality in those with high BMI, deaths from respiratory and other noncardiovascular causes were more prominent in individuals with low BMI [5].

Diabetes is a well‐established risk factor for increased vulnerability to infectious respiratory diseases, including pneumonia, pulmonary tuberculosis (TB) and COVID‐19 [6, 7, 8]. Compared to people without diabetes, patients with type 2 diabetes (T2DM) face substantially higher infection‐related mortality, with infections accounting for approximately 13% of all deaths—ranking third after CVD and cancer [9]. Among infection‐related causes, lower respiratory tract infections, especially pneumonia, contribute significantly to this excess mortality [9].

While general population research has shown an association between low BMI and increased mortality from communicable diseases [10]—especially respiratory infections—data focusing on individuals with diabetes are limited. Individuals with diabetes who are underweight may be especially susceptible to severe infection due to reduced muscle mass, impaired nutritional status and compromised immune function [11]. Although obesity often garners attention in discussions of poor outcomes in diabetes [12, 13], underweight status is an underrecognized but critical determinant of infection‐related vulnerability.

In this study, we aimed to examine the association between underweight status and mortality from respiratory infections, including pneumonia, pulmonary TB and COVID‐19, in a nationwide cohort of individuals with diabetes

## 2 Methods

### 2.1 Data Source

This study analyzed data from the Korean National Health Insurance Service (NHIS), the sole public insurer that administers South Korea's National Health Insurance program, covering nearly the entire population of approximately 50 million individuals. As enrollment is mandatory, the NHIS database serves as a nationally representative cohort. The dataset has been comprehensively described in prior studies [14].

The NHIS conducts a biennial health examination program for individuals aged 20 years and older, collecting detailed health‐related information, including anthropometric measurements (height, weight and waist circumference), blood pressure, lifestyle factors (smoking status, alcohol consumption and physical activity) and laboratory test results (fasting glucose, lipid profiles, complete blood counts, as well as liver and renal function tests).

### 2.2 Study Population

This study included adults (≥ 20 years) diagnosed with T2DM who underwent NHIS routine health screenings between 1 January 2015 and 31 December 2016. Participants were followed until 31 December 2022. T2DM was defined as a fasting blood glucose level of ≥ 126 mg/dL or the presence of International Classification of Diseases, 10th Revision (ICD‐10) codes E11–E14 with a documented history of antidiabetic medication use before the screening date.

Of the initial 2 617 160 individuals identified, we excluded those younger than 20 years (*n* = 323), those with missing key variables (*n* = 88 568) and those who died within 1 year of their screening date (*n* = 19 860) to account for potential reverse causality. After these exclusions, a total of 2 508 409 participants were included in the final analysis (Figure S1).

This study was approved by the Institutional Review Board of the Hallym University Dongtan Sacred Heart Hospital (HDT 2025‐02‐001). Given the use of anonymized and de‐identified data, the requirement for informed consent was waived

### 2.3 Definition of Underweight and BMI Classification

BMI categories were defined as follows: underweight was classified as BMI < 18.5 kg/m^2^ and further divided into mildly underweight (BMI = 17.0–18.4 kg/m^2^), moderately underweight (BMI = 16.0–16.9 kg/m^2^) and severely underweight (BMI < 16.0 kg/m^2^) [15]. For individuals with BMI ≥ 18.5 kg/m^2^, the Asia‐Pacific criteria were used to define normal weight (18.5–22.9 kg/m^2^), overweight (23.0–24.9 kg/m^2^), obesity class I (25.0–29.9 kg/m^2^) and obesity class II (≥ 30.0 kg/m^2^). For more detailed comparison, we divided class II as BMI of 30.0–34.9 and ≥ 35.0 kg/m^2^ (obesity class III) [16].

To evaluate the robustness of associations between malnutrition severity and respiratory infection‐related mortality, we additionally applied the Global Leadership Initiative on Malnutrition (GLIM) phenotypic BMI thresholds to classify nutritional status [17]. Participants were categorized as having normal nutritional status, stage 1 malnutrition (BMI < 20 kg/m^2^ for age < 70 years or < 22 kg/m^2^ for age ≥ 70 years) or stage 2 malnutrition (BMI < 18.5 kg/m^2^ for age < 70 years or < 20 kg/m^2^ for age ≥ 70 years).

### 2.4 Mortality Outcome

Mortality due to respiratory infection‐related causes was assessed. Mortality due to respiratory tract infections (including influenza, pneumonia and empyema) was those coded as J00–J06, J09–J18, J20–J22, J85–J86, mortality due to TB under the code A15–A19 and mortality due to COVID‐19 U07.

### 2.5 Statistical Analysis

Baseline characteristics of the study population are summarized according to the BMI categories. Hazard ratios (HRs) and 95% confidence intervals (CIs) for respiratory infection‐related mortality across BMI groups were estimated using Cox proportional hazards regression models. For analyses focused on underweight, the reference group was defined as individuals with BMI ≥ 18.5 kg/m^2^. In analyses covering the full BMI spectrum, including obesity categories, the reference group was set at BMI 25.0–29.9 kg/m^2^. To facilitate interpretation across standard Asian BMI classifications, additional analyses were conducted across the full BMI spectrum using normal weight (BMI = 18.5–22.9 kg/m^2^) as the reference group, and these results are presented in the Supplementary Materials. Survival curves were generated using the Kaplan–Meier method. Associations between outcomes and BMI as a continuous variable were assessed using penalized spline regression.

Multivariable models were sequentially adjusted for potential confounders. Model 1 was unadjusted. Model 2 was adjusted for age and sex. Model 3 was further adjusted for socioeconomic factors (income level), lifestyle behaviours (smoking, alcohol consumption and physical activity), clinical comorbidities (hypertension, dyslipidemia, chronic kidney disease [CKD], chronic heart failure, chronic obstructive pulmonary disease [COPD], chronic respiratory failure and active cancer diagnosed within 5 years) and diabetes‐related factors (fasting glucose levels, use of ≥ 3 oral antidiabetic medications or insulin and diabetes duration).

Stratified analyses were conducted to explore potential effect modification by age, sex and comorbidity status. Interaction terms were tested using Wald tests.

In the primary analysis, deaths occurring within the first year of follow‐up were excluded to minimize potential reverse causation. Given the acute and often rapidly progressive nature of respiratory infections, the application of a lag time may be less critical for respiratory mortality outcomes than for chronic disease endpoints. Therefore, this analysis was repeated without excluding early deaths. Sensitivity analyses were conducted using Fine–Grey subdistribution hazard models to account for competing risks of death from CVD, cancer and diabetes‐specific causes. This approach acknowledges that deaths from other causes preclude the occurrence of respiratory infection–related death.

Additional robustness checks included reclassification of participants according to GLIM phenotypic BMI thresholds (normal nutritional status, stage 1 malnutrition and stage 2 malnutrition) and restriction of the analysis to participants aged ≥ 65 years.

All statistical analyses were performed using SAS Version 9.4 (SAS Institute Inc., Cary, NC, USA). A two‐sided *p* value < 0.05 was considered statistically significant.

## 3 Results

### 3.1 Baseline Characteristics of the Study Participants

The study included 2 508 409 participants with T2DM, with a mean age of 59.6 ± 12.0 years (Table 1, Table S1). Of the total population, 34.5% were aged 65 years or older (Table S2). Regarding smoking status, 22.5% were current smokers, 22.5% were former smokers, and 55.0% had never smoked.

**TABLE 1:** Baseline characteristics of the participants stratified by underweight status.

Variable	Total	BMI group					
		Underweight			Nonunderweight		
		< 16 (Severe)	16–16.9 (Moderate)	17–18.4 (Mild)	≥ 18.5	*p*	*p* for trend
*n*	2 508 409	2571	5445	26 157	2 474 236		
BMI (kg/m^2^)	25.3 ± 3.5	15.1 ± 0.8	16.5 ± 0.3	17.9 ± 0.4	25.4 ± 3.4	< 0.001	< 0.001
Weight (kg)	67.4 ± 12.6	38.6 ± 5.1	42.6 ± 5.1	46.4 ± 5.4	67.7 ± 12.4	< 0.001	< 0.001
Height (cm)	162.8 ± 9.4	159.5 ± 9.7	160.3 ± 9.4	160.9 ± 9.2	162.8 ± 9.4	< 0.001	< 0.001
Waist circumference (cm)	86.2 ± 9.0	66.0 ± 7.5	67.7 ± 6.4	70.1 ± 5.9	86.4 ± 8.8	< 0.001	< 0.001
Central obesity, *n* (%)	1 032 090 (41.2)	41 (1.6)	54 (1.0)	254 (1.0)	1 031 741 (41.7)	< 0.001	< 0.001
Age (years)	59.6 ± 12.0	65.4 ± 15.3	63.4 ± 15.0	62.1 ± 14.2	59.6 ± 12.0	< 0.001	< 0.001
≥ 65 years, *n* (%)	864 687 (34.5)	1372 (53.4)	2614 (48.0)	11 536 (44.1)	849 165 (34.3)	< 0.001	< 0.001
Male, *n* (%)	1 510 820 (60.2)	1274 (49.6)	2855 (52.4)	13 993 (53.5)	1 492 698 (60.3)	< 0.001	< 0.001
Systolic BP (mmHg)	128.5 ± 15.1	119.5 ± 17.1	120.5 ± 16.9	121.9 ± 16.5	128.6 ± 15.0	< 0.001	< 0.001
Diastolic BP (mmHg)	78.1 ± 10.0	73.6 ± 10.7	73.9 ± 10.6	74.2 ± 10.3	78.1 ± 9.9	< 0.001	< 0.001
Fasting glucose (mg/dL)	144.5 ± 45.8	153.9 ± 75.3	150.7 ± 68.4	149.0 ± 62.7	144.5 ± 45.5	< 0.001	< 0.001
Total cholesterol (mg/dL)	185.2 ± 43.7	176.2 ± 42.2	176.5 ± 41.6	177.2 ± 41.0	185.3 ± 43.8	< 0.001	< 0.001
Triglyceride (mg/dL)^a^	137.7 (137.6, 137.8)	96.4 (94.6, 98.3)	95.1 (93.7, 96.4)	95.5 (94.9, 96.1)	138.4 (138.3, 138.5)	< 0.001	< 0.001
HDL‐cholesterol (mg/dL)	51.0 ± 14.7	59.7 ± 19.1	59.9 ± 21.5	58.9 ± 17.5	50.8 ± 14.6	< 0.001	< 0.001
LDL‐cholesterol (mg/dL)	103.1 ± 38.4	94.5 ± 36.3	94.8 ± 35.6	96.1 ± 35.9	103.2 ± 38.4	< 0.001	< 0.001
AST (IU/L)	29.9 ± 22.2	33.3 ± 39.0	32.6 ± 43.4	30.6 ± 37.9	29.9 ± 21.9	< 0.001	< 0.001
ALT (IU/L)	31.9 ± 26.8	23.3 ± 29.3	23.5 ± 27.5	23.1 ± 25.9	32.0 ± 26.7	< 0.001	< 0.001
eGFR (mL/min/1.73m^2^)	84.1 ± 20.2	82.6 ± 22.7	83.8 ± 22.7	84.6 ± 21.9	84.1 ± 20.2	< 0.001	0.9402
*Social history*							
Smoking, *n* (%)						< 0.001	—
Never smoker	1 380 292 (55.0)	1562 (60.8)	3178 (58.4)	15 060 (57.6)	1 360 492 (55.0)		
Ex‐smoker	563 329 (22.5)	299 (11.6)	710 (13.0)	3689 (14.1)	558 631 (22.6)		
Current smoker	564 788 (22.5)	710 (27.6)	1557 (28.6)	7408 (28.3)	555 113 (22.4)		
Alcohol, *n* (%)						< 0.001	—
None	1 450 856 (57.8)	1886 (73.4)	3822 (70.2)	17 409 (66.6)	1 427 739 (57.7)		
Mild	829 920 (33.1)	508 (19.8)	1252 (23.0)	6817 (26.1)	821 343 (33.2)		
Heavy	227 633 (9.1)	177 (6.9)	371 (6.8)	1931 (7.4)	225 154 (9.1)		
Regular exercise, *n* (%)	543 953 (21.7)	264 (10.3)	817 (15.0)	4628 (17.7)	538 244 (21.8)	< 0.001	< 0.001
Low income, *n* (%)	540 538 (21.6)	732 (28.5)	1487 (27.3)	6762 (25.9)	531 557 (21.5)	< 0.001	< 0.001
*Medical history*							
Diabetes duration (years)						< 0.001	—
New onset	758 219 (30.2)	871 (33.9)	1767 (32.5)	8122 (31.1)	747 459 (30.2)		
< 5 years	594 116 (23.7)	491 (19.1)	982 (18.0)	4690 (17.9)	587 953 (23.8)		
< 10 years	522 520 (20.8)	496 (19.3)	1012 (18.6)	4919 (18.8)	516 093 (20.9)		
≥ 10 years	633 554 (25.3)	713 (27.7)	1684 (30.9)	8426 (32.2)	622 731 (25.2)		
≥ 3 oral antidiabetic drugs, *n* (%)	563 143 (22.5)	455 (17.7)	1115 (20.5)	5570 (21.3)	556 003 (22.5)	< 0.001	< 0.001
Insulin use, *n* (%)	205 191 (8.2)	371 (14.4)	818 (15.0)	3607 (13.8)	200 395 (8.1)	< 0.001	< 0.001
Hypertension, *n* (%)	1 477 384 (58.9)	1038 (40.4)	2220 (40.8)	10 490 (40.1)	1 463 636 (59.2)	< 0.001	< 0.001
Dyslipidemia, *n* (%)	1 436 734 (57.3)	842 (32.8)	1825 (33.5)	9776 (37.4)	1 424 291 (57.6)	< 0.001	< 0.001
Chronic kidney disease, *n* (%)	274 356 (10.9)	418 (16.3)	795 (14.6)	3338 (12.8)	269 805 (10.9)	< 0.001	< 0.001
History of CVD, *n* (%)	269 723 (10.8)	459 (17.9)	806 (14.8)	3352 (12.8)	265 106 (10.7)	< 0.001	< 0.001
Chronic heart failure, *n* (%)	148 103 (5.9)	222 (8.6)	433 (7.9)	1686 (6.5)	145 762 (5.9)	< 0.001	< 0.001
COPD, *n* (%)	171 383 (6.8)	485 (18.9)	887 (16.3)	3124 (11.9)	166 887 (6.7)	< 0.001	< 0.001
Chronic respiratory failure, *n* (%)	217 (0.0)	6 (0.2)	3 (0.1)	11 (0.0)	197 (0.0)	< 0.001	< 0.001
Active cancer, *n* (%)^b^	111 837 (4.5)	192 (7.5)	461 (8.5)	1923 (7.4)	109 261 (4.4)	< 0.001	< 0.001
Active lung cancer, *n* (%)^b^	6010 (0.2)	13 (0.5)	25 (0.5)	106 (0.4)	5866 (0.2)	< 0.001	< 0.001

*Note:* Data are presented as mean ± standard deviation (SD) for continuous variables or *n* (%) for categorical variables.

Abbreviations: ALT, alanine aminotransferase; AST, aspartate aminotransferase; BMI, body mass index; CKD, chronic kidney disease; COPD, chronic obstructive pulmonary disease; CVD, cardiovascular disease; DBP, diastolic blood pressure; eGFR, estimated glomerular filtration rate; HDL, high‐density lipoprotein; LDL, low‐density lipoprotein; SBP, systolic blood pressure.

^a^ Triglycerides are presented as geometric mean (95% confidence interval) after log transformation due to right‐skewed distribution.

^b^ Active cancer and active lung cancer were defined as those diagnosed within 5 years prior to study enrollment.

A total of 34 173 individuals (1.36%) were underweight, classified into the following three subgroups: mildly underweight (BMI = 17.0–18.4 kg/m^2^, 26 157 [1.04%]), moderately underweight (BMI = 16.0–16.9 kg/m^2^, 5445 [0.22%]) and severely underweight (BMI < 16.0 kg/m^2^, 2571 [0.10%]).

The distribution of diabetes duration was balanced across the cohort: 30.2% were newly diagnosed, 23.7% had diabetes for less than 5 years, 20.8% for 5–10 years and 25.3% for more than 10 years. Regarding treatment, 22.5% of participants were on three or more oral antidiabetic agents, and 8.2% were receiving insulin therapy. A higher proportion of underweight individuals were on insulin, and they also exhibited higher fasting glucose levels compared to those in higher BMI categories.

Participants in the underweight group had a greater burden of comorbidities, including higher prevalence rates of CKD, CVD, COPD, chronic respiratory failure and active cancer diagnosed within 5 years. Conversely, individuals with BMI ≥ 35.0 kg/m^2^ exhibited the highest rates of hypertension (74.6%), dyslipidemia (58.6%) and abdominal obesity (99.1%).

### 3.2 Association Between Underweight Status and Respiratory Infection Mortality in Diabetes

During a median follow‐up of 6 years (interquartile range, 5.34–6.33), a total of 18 024 (0.72%) deaths occurred from respiratory infections among 2 508 409 individuals with T2DM. This included 12 703 cases of death from influenza/pneumonia, 808 cases of death from TB and 4513 cases of death from COVID‐19. The risk of mortality from respiratory diseases was significantly higher in underweight individuals, with a stepwise increase observed according to the severity of underweight (incidence ratios = 16.78, 10.06, 5.84 and 1.18 per 1000 person‐years for severe, moderate, mild underweight and nonunderweight, respectively) (Table 2). In the unadjusted model, the risk of death due to respiratory infections was 15.5 times higher in individuals with severe underweight compared to nonunderweight. Even after full adjustment for potential confounders, the risk remained more than seven times higher. Compared to those with a BMI of 18.5 kg/m^2^ or higher, individuals in the severely underweight group (aHR, 7.27; 95% CI, 6.31–8.38) exhibited the highest mortality risk, followed by those in the moderately (aHR, 4.79; 95% CI, 4.25–5.41) and mildly (aHR, 3.15; 95% CI, 2.93–3.38) underweight groups.

**TABLE 2:** Risk of mortality from respiratory diseases in underweight individuals with diabetes.

Outcome	BMI group	N	Event	Duration	IR per 1000	Model 1	Model 2	Model 3
All deaths due to	< 16	2571	196	11 680	16.8	15.46 (13.43–17.80)	7.39 (6.41–8.51)	7.27 (6.31–8.38)
Respiratory diseases	16–16.9	5445	271	26 933	10.1	9.01 (7.99–10.16)	5.05 (4.48–5.70)	4.79 (4.25–5.41)
	17–18.4	26 157	804	137 653	5.8	5.11 (4.76–5.49)	3.29 (3.07–3.54)	3.15 (2.93–3.38)
	≥ 18.5	2 474 236	16 753	14 254 791	1.2	1 (ref.)	1 (ref.)	1 (ref.)
	*p* value					< 0.001	< 0.001	< 0.001
	*p* for trend					< 0.001	< 0.001	< 0.001
Influenza/pneumonia	< 16	2571	158	11 680	13.5	17.29 (14.78–20.23)	7.66 (6.54–8.96)	7.31 (6.24–8.57)
	16–16.9	5445	227	26 933	8.4	10.60 (9.29–12.08)	5.56 (4.87–6.35)	5.11 (4.47–5.83)
	17–18.4	26 157	627	137 653	4.6	5.65 (5.21–6.12)	3.47 (3.20–3.76)	3.23 (2.98–3.51)
	≥ 18.5	2 474 236	11 691	14 254 791	0.8	1 (ref.)	1 (ref.)	1 (ref.)
	*p* value					< 0.001	< 0.001	< 0.001
	*p* for trend					< 0.001	< 0.001	< 0.001
Tuberculosis	< 16	2571	15	11 680	1.3	25.60 (15.35–42.69)	12.19 (7.28–20.41)	9.93 (5.91–16.69)
	16–16.9	5445	13	26 933	0.5	9.59 (5.54–16.59)	5.30 (3.06–9.19)	4.34 (2.50–7.55)
	17–18.4	26 157	57	137 653	0.4	8.19 (6.26–10.73)	5.21 (3.97–6.84)	4.52 (3.43–5.95)
	≥ 18.5	2 474 236	723	14 254 791	0.1	1 (ref.)	1 (ref.)	1 (ref.)
	*p* value					< 0.001	< 0.001	< 0.001
	*p* for trend					< 0.001	< 0.001	< 0.001
COVID‐19	< 16	2571	23	11 680	2.0	7.83 (5.20–11.79)	4.70 (3.12–7.08)	5.16 (3.42–7.79)
	16–16.9		31	26 933	1.2	4.26 (2.99–6.06)	2.89 (2.03–4.11)	3.10 (2.17–4.41)
	17–18.4	26 157	120	137 653	0.9	3.06 (2.55–3.66)	2.25 (1.88–2.70)	2.35 (1.96–2.83)
	≥ 18.5	2 474 236	4339	14 254 791	0.3	1 (ref.)	1 (ref.)	1 (ref.)
	*p* value					< 0.001	< 0.001	< 0.001
	*p* for trend					< 0.001	< 0.001	< 0.001

*Note:* Model 1: Unadjusted. Model 2: Adjusted for age and sex. Model 3: Adjusted for age, sex, income, smoking status, alcohol intake, physical activities, the presence of hypertension, dyslipidemia, chronic kidney disease, chronic heart failure, chronic obstructive pulmonary disease, chronic respiratory failure, active cancer diagnosed within 5 years, fasting glucose, use of three or more oral antidiabetic medication or insulin and duration of diabetes.

**FIGURE 1:**
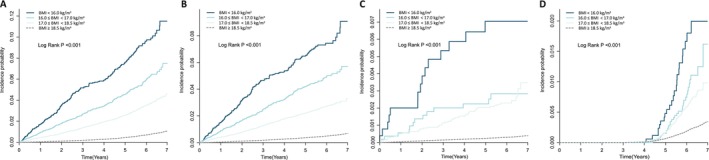
**Cumulative incidence of respiratory infection‐related mortality by underweight severity in individuals with type 2 diabetes.** Kaplan–Meier survival curves showing cumulative incidence of (A) total respiratory infection‐related mortality, (B) influenza and pneumonia mortality, (C) tuberculosis mortality, and (D) COVID‐19 mortality according to baseline BMI categories. Individuals were grouped as follows: severe underweight (BMI < 16.0 kg/m^2^), moderate underweight (BMI = 16.0–16.9 kg/m^2^), mild underweight (BMI = 17.0–18.4 kg/m^2^) and nonunderweight (BMI ≥ 18.5 kg/m^2^). Log‐rank *P* values for overall differences across groups are shown in each panel. Incidence probability was estimated over a median follow‐up of 6 years. BMI, body mass index.

A similar pattern was observed across specific causes of respiratory mortality. The adjusted HR for influenza and pneumonia mortality was 7.31 (95% CI, 6.24–8.57) in the severely underweight group. Mortality risk was also markedly increased for TB (aHR, 9.93; 95% CI, 5.91–16.69) and COVID‐19 (aHR, 5.16; 95% CI, 3.42–7.79) (Table 2, Figure 1).

**FIGURE 2:**
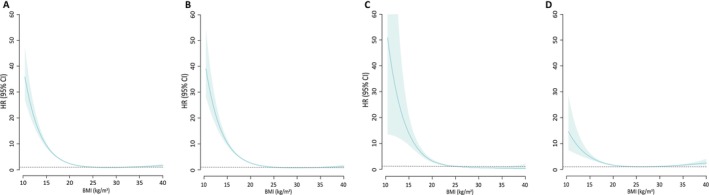
**Adjusted hazard ratios for respiratory infection‐related mortality across the full spectrum of BMI in individuals with type 2 diabetes.** Restricted cubic spline models were used to assess the continuous relationship between BMI and mortality risk, with BMI 25.0 kg/m^2^ as the reference. Hazard ratios (HRs) and 95% confidence intervals (CIs) were estimated using Cox proportional hazards models adjusted for age, sex, income, smoking status, alcohol intake, physical activities, the presence of hypertension, dyslipidemia, chronic kidney disease, chronic heart failure, chronic obstructive pulmonary disease, chronic respiratory failure, active cancer diagnosed within 5 years, fasting glucose, use of three or more oral antidiabetic medication or insulin and duration of diabetes. (A) All respiratory infection‐related deaths, (B) influenza and pneumonia, (C) tuberculosis and (D) COVID‐19.

Across the full BMI spectrum (reference: 18.0–29.9 kg/m^2^), mortality among individuals with underweight increased sharply with decreasing BMI (Table S3, Figure 2). Obesity (BMI = 30.0–34.9 kg/m^2^) was only modestly associated with elevated respiratory mortality, and when specific causes were evaluated, only COVID‐19 mortality showed a significant increase (aHR, 1.28; 95% CI, 1.13–1.46). For BMI ≥ 35.0 kg/m^2^, mortality was significantly increased for all respiratory infections combined (aHR, 1.69; 95% CI, 1.38–2.07), influenza/pneumonia (aHR, 1.52; 95% CI, 1.15–2.00) and COVID‐19 (aHR, 1.97; 95% CI, 1.44–2.70), but not for TB. Notably, the magnitude of mortality risk in obesity was substantially lower than in any underweight category.

When the normal‐weight group (BMI = 18.5–22.9 kg/m^2^) was used as the reference, most higher BMI categories were associated with lower respiratory infection–related mortality until BMI reached ≥ 35.0 kg/m^2^ (Table S4). At BMI ≥ 35 kg/m^2^, COVID‐19 mortality was significantly elevated (aHR, 1.48; 95% CI, 1.08–2.03).

### 3.3 Subgroup Analysis

**FIGURE 3:**
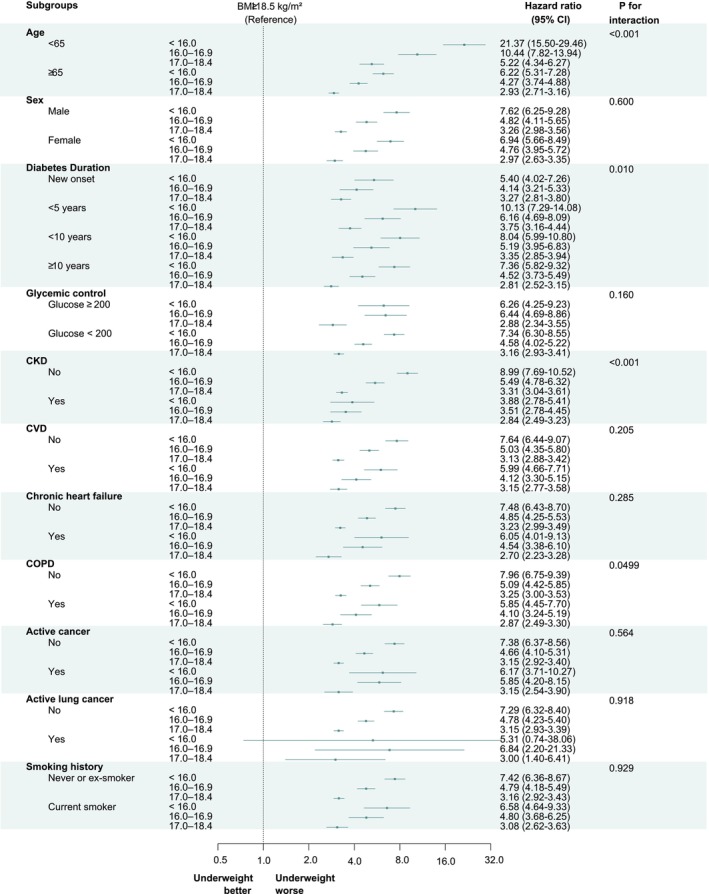
**Subgroup analyses of the association between underweight status and respiratory infection‐related mortality in individuals with type 2 diabetes.** Forest plots showing adjusted hazard ratios (HRs) and 95% confidence intervals (CIs) for respiratory mortality by severity of underweight status (BMI < 18.5 kg/m^2^) across clinically relevant subgroups. BMI ≥ 18.5 kg/m^2^ served as the reference group. Subgroups included age, sex, diabetes duration, glycemic control (fasting glucose ≥ 200 vs. < 200 mg/dL), smoking history, chronic obstructive pulmonary disease (COPD), chronic kidney disease (CKD), cardiovascular disease (CVD), chronic heart failure, any active cancer and active lung cancer.

In the stratified analysis, underweight status was consistently associated with significantly elevated respiratory mortality among individuals with diabetes, with certain subgroups demonstrating more pronounced risks (Table S5, Figure 3).

The association was strongest among individuals aged < 65 years, who exhibited a markedly higher risk of respiratory mortality compared to those aged ≥ 65 years (*P* for interaction < 0.001). Importantly, in analyses restricted to individuals aged ≥ 65 years, underweight status remained significantly associated with increased respiratory infection–related mortality (Table S6).

When stratified by diabetes duration, individuals with pre‐existing T2DM exhibited a significantly greater increase in respiratory mortality associated with underweight status compared to those with new‐onset T2DM (*P* for interaction = 0.010). However, poor glycemic control, as defined by fasting glucose ≥ 200 mg/dL, did not further amplify the mortality risk among underweight individuals.

Interestingly, underweight individuals without comorbid COPD or prior CKD experienced even greater elevations in respiratory mortality compared to those with these chronic conditions. Active cancer diagnosed within 5 years, including lung cancer, did not modify the association between underweight and respiratory mortality, and similarly, smoking status was not significantly associated with differential respiratory mortality risk among underweight individuals with diabetes.

### 3.4 Sensitivity Analysis

The results remained robust when the 1‐year lag period was removed, with underweight status continuing to demonstrate a markedly elevated risk for respiratory mortality across all categories (Table S7). Specifically, the aHR for all deaths due to respiratory diseases was 8.36 (95% CI, 7.38–9.46) for people who were severely underweight, closely aligning with the primary analysis. Cause‐specific mortality risks for people who are severely underweight were similarly elevated for influenza and pneumonia (aHR, 8.28; 95% CI, 7.21–9.51) and TB (aHR, 13.48; 95% CI, 9.00–20.26). The association with COVID‐19–related mortality remained unchanged (aHR, 5.16; 95% CI, 3.42–7.79), as COVID‐19 deaths only occurred during the post‐2019 follow‐up period.

In competing risks analyses accounting for deaths due to CVD, diabetes and cancer, underweight status remained a strong independent predictor of respiratory mortality. Severely underweight individuals experienced significantly higher hazards for all‐cause respiratory mortality (aHR, 5.83; 95% CI, 4.99–6.82). This pattern persisted across all cause‐specific respiratory deaths, with the greatest risk observed for TB (aHR, 8.53; 95% CI, 5.01–14.52), followed by influenza and pneumonia (aHR, 6.05; 95% CI, 5.09–7.18) and COVID‐19 (aHR, 3.71; 95% CI, 2.44–5.62) (Table S8).

In sensitivity analyses using GLIM‐based phenotypic BMI thresholds, participants were reclassified into normal nutritional status, stage 1 malnutrition and stage 2 malnutrition (Table S9, Figure S2). A clear graded association was observed, with progressively higher risks of respiratory infection–related mortality across worsening malnutrition stages. Compared with individuals with normal nutritional status, those with stage 2 malnutrition had the highest mortality from all respiratory diseases (aHR, 3.32; 95% CI, 3.18–3.47), followed by those with stage 1 malnutrition (aHR, 1.78; 95% CI, 1.71–1.86). Similar dose–response patterns were observed for influenza/pneumonia, TB and COVID‐19 (Table S10). Because GLIM criteria incorporate age‐specific BMI thresholds, we further conducted analyses restricted to individuals aged ≥ 65 years (Table S11). In this older subgroup, the graded association persisted.

## 4 Discussion

In this large, population‐based cohort study of individuals with diabetes, underweight status was strongly and consistently associated with elevated mortality from respiratory infections. This association was evident not only for all respiratory infection‐related deaths but also across specific causes, including influenza and pneumonia, TB and COVID‐19. Among these, TB‐related mortality showed the greatest increase in underweight individuals. Notably, even mild underweight status conferred a higher mortality risk than severe obesity (BMI ≥ 35 kg/m^2^). Although underweight individuals comprised a small proportion of the diabetic population, these findings emphasize underweight status as an important, yet often underrecognized, risk factor for respiratory mortality in people with diabetes.

Patients with diabetes are already predisposed to respiratory infections due to impaired pulmonary function and immune dysregulation [18, 19, 20, 21]. Prior research in Korea found that severe underweight status among patients hospitalized with pneumonia was independently associated with higher 30‐day mortality [22]. In our study, underweight individuals with diabetes appeared particularly vulnerable, with influenza and pneumonia‐related mortality rising sharply as underweight severity increased. These findings align with UK data showing a 2.3‐fold higher risk of influenza‐associated pneumonia in underweight individuals with diabetes compared to nondiabetic, normal‐weight counterparts [23]. By contrast, a Japanese study that used a binary BMI cutoff (< 25 vs. ≥ 25 kg/m^2^) did not detect a significant interaction between BMI and diabetes for respiratory mortality, possibly missing more granular associations [24]. In our study, a clear dose–response was observed, with the highest mortality in those severely underweight (BMI < 16 kg/m^2^).

Underweight individuals with diabetes also showed markedly elevated TB mortality. This likely reflects a synergy between diabetes‐induced impairments in monocyte and macrophage function, reduced T‐cell proliferation, diminished interferon‐gamma production and the immunosuppressive effects of malnutrition [25, 26, 27, 28]. Notably, in our findings, elevated TB‐related mortality was observed only in underweight individuals with diabetes, whereas even those with severe obesity did not exhibit an increased mortality risk.

We also observed significantly increased COVID‐19 mortality in underweight individuals with diabetes, particularly those with BMI < 16 kg/m^2^. While those with obesity (BMI ≥ 30 kg/m^2^) also had increased mortality, the risk was notably high among the severely underweight, consistent with a J‐shaped relationship between BMI and COVID‐19 severity. Diabetes is a well‐established risk factor for COVID‐19 susceptibility and severity, with meta‐analyses reporting a 2.3‐fold higher mortality risk than individuals without diabetes [29, 30, 31]. Although increasing BMI is generally associated with worse COVID‐19 outcomes, several studies have identified a J‐shaped curve, with elevated risks at both low (≤ 20 kg/m^2^) and high (> 28 kg/m^2^) BMI levels [32]. Interestingly, the positive association of BMI and mortality appears attenuated in people with T2DM, suggesting that metabolic vulnerability may be more critical than BMI alone [32]. Supporting this, a study on older adults in South Korea identified underweight status as an independent predictor of COVID‐19 mortality, while being overweight was paradoxically protective [33].

Underweight status (BMI < 18.5 kg/m^2^) has previously been associated with more than a twofold increase in infection‐related hospitalization and death, independent of diabetes [2]. COPD represents a major comorbidity among underweight patients with diabetes and independently predicts respiratory mortality [34]. Low BMI frequently coexists with advanced COPD due to cachexia and muscle wasting [35, 36]. Although some underweight individuals may have underlying wasting conditions, our stratified analyses showed that even those without COPD or CKD experienced significantly elevated respiratory mortality, suggesting that low body weight itself confers an independent risk. These findings were consistent across sensitivity analyses, including the models excluding and including early deaths. The risk was most pronounced in individuals under 65 years, aligning with a previous UK finding that younger adults with diabetes face nearly fourfold higher infection‐related mortality compared to the general population [9, 37]. These premature deaths have considerable social and economic implications.

This study has several strengths. It leveraged a large, nationally representative cohort of individuals with T2DM and included a broad range of BMI values—particularly at the lower end of the spectrum—providing insights rarely captured in Western populations [5]. The application of detailed stratification, competing risk models and lag‐time sensitivity analyses strengthens the reliability of the findings. Additionally, subgroup analyses helped identify particularly vulnerable populations, such as younger adults and those without major comorbidities. Importantly, the observed associations were robust across multiple sensitivity analyses, including alternative nutritional classifications based on GLIM phenotypic criteria. Similar graded associations were also observed when analyses were restricted to adults aged ≥ 65 years, supporting the consistency of our findings in older populations.

Nevertheless, several limitations should be considered. BMI was used as a proxy for nutritional status and body composition, which may not fully reflect underlying physiologic vulnerability. BMI should therefore not be interpreted as a direct causal factor for respiratory infection–related mortality. Rather, underweight status likely serves as a surrogate marker for underlying wasting‐related conditions, including malnutrition, sarcopenia, frailty and chronic disease burden, which collectively increase susceptibility to severe and fatal infections. Consistent results obtained using GLIM‐based phenotypic malnutrition classifications further support the interpretation that the excess respiratory mortality observed in underweight individuals reflects underlying wasting‐related vulnerability rather than BMI per se. Despite adherence to national standards for cause‐of‐death classification, some misclassification may have occurred—particularly between overlapping infectious causes such as pneumonia and COVID‐19. RSV‐specific mortality could not be separately examined. Moreover, while our research benefits from a large, representative nationwide cohort, caution is needed when generalizing these findings to populations with differing genetic profiles, healthcare infrastructure or environmental exposures. The NHIS database also lacks comprehensive glycemic measures such as HbA1c, as well as serum albumin, inflammatory markers (e.g., C‐reactive protein) and information on systemic glucocorticoid use, precluding adjustment for these potentially relevant factors.

In conclusion, underweight status is a strong and independent predictor of respiratory infection‐related mortality in individuals with diabetes, with even mild underweight carrying greater mortality risks than severe obesity (BMI ≥ 35 kg/m^2^) (Graphical Abstract). This elevated risk spans a spectrum of respiratory infectious diseases—including influenza, pneumonia, TB and COVID‐19—and is especially pronounced in younger adults and those without major comorbidities. These findings underscore the importance of recognizing low body weight as a clinically relevant risk factor in diabetes care.

## Funding

This research was supported by a grant from the Korea Health Technology R&D Project through the Korea Health Industry Development Institute (KHIDI), funded by the Ministry of Health & Welfare, Republic of Korea (Grant Number: HBI23C0679). The funding source had no role in the study design, data collection, analysis, interpretation or writing of the manuscript.

## Conflicts of Interest

The authors declare no conflicts of interest.

## Data Sharing

The data used in this study were obtained from the Korean NHIS database. Access to these data is available upon reasonable request and approval from the Korean NHIS. Due to privacy regulations and data sharing policies, the data cannot be made publicly available.

## Supporting information


**Figure S1:** Cohort derivation flowchart.
**Figure S2:** Cumulative incidence of respiratory infection‐related mortality by GLIM‐based malnutrition staging in individuals with type 2 diabetes.
**Table S1:** Baseline characteristics of the participants stratified by underweight status.
**Table S2:** Baseline characteristics of participants aged ≥ 65 years stratified by underweight status.
**Table S3:** Risk of mortality from respiratory diseases across the BMI spectrum (reference: BMI = 25.0–29.9 kg/m2).
**Table S4:** Risk of mortality from respiratory diseases across the BMI spectrum (reference: BMI = 18.5–22.9 kg/m2).
**Table S5:** Subgroup analysis for the risk of mortality from respiratory infection.
**Table S6:** Risk of mortality from respiratory diseases according to BMI categories among individuals aged ≥ 65 years with diabetes.
**Table S7:** Sensitivity analysis excluding the 1‐year lag period.
**Table S8:** Sensitivity analysis with cardiovascular, diabetes and cancer mortality as competing risks.
**Table S9:** Baseline characteristics of participants according to GLIM‐defined malnutrition stage.
**Table S10:** Risk of respiratory disease–related mortality according to GLIM‐defined malnutrition stage in individuals with diabetes.
**Table S11:** Risk of respiratory disease–related mortality according to GLIM‐defined malnutrition stage among individuals aged ≥ 65 years with diabetes.
