# Supplementary material for: Underweight Status Amplifies Respiratory Infection Mortality in Diabetes: Findings From a Nationwide Cohort Study
Source: J Cachexia Sarcopenia Muscle. 2026 Mar 28;17(2):e70263. doi: 10.1002/jcsm.70263 (PMC13054675; doi:10.1002/jcsm.70263)
Supplement: Supplementary file 1 — Figure S1: Cohort derivation flowchart. Figure S2: Cumulative incidence of respiratory infection‐related mortality by GLIM‐based malnutrition staging in individuals with type 2 diabetes. Table S1: Baseline characteristics of the participants stratified by underweight status. Table S2: Baseline characteristics of participants aged ≥ 65 years stratified by underweight status. Table S3: Risk of mortality from respiratory diseases across the BMI spectrum (reference: BMI = 25.0–29.9 kg/m2). Table S4: Risk of mortality from respiratory diseases across the BMI spectrum (reference: BMI = 18.5–22.9 kg/m2). Table S5: Subgroup analysis for the risk of mortality from respiratory infection. Table S6: Risk of mortality from respiratory diseases according to BMI categories among individuals aged ≥ 65 years with diabetes. Table S7: Sensitivity analysis excluding the 1‐year lag period. Table S8: Sensitivity analysis with cardiovascular, diabetes and cancer mortality as competing risks. Table S9: Baseline characteristics of participants according to GLIM‐defined malnutrition stage. Table S10: Risk of respiratory disease–related mortality according to GLIM‐defined malnutrition stage in individuals with diabetes. Table S11: Risk of respiratory disease–related mortality according to GLIM‐defined malnutrition stage among individuals aged ≥ 65 years with diabetes. [file JCSM-17-e70263-s001.pdf]

Supplementary Figure 1. Cohort derivation flowchart

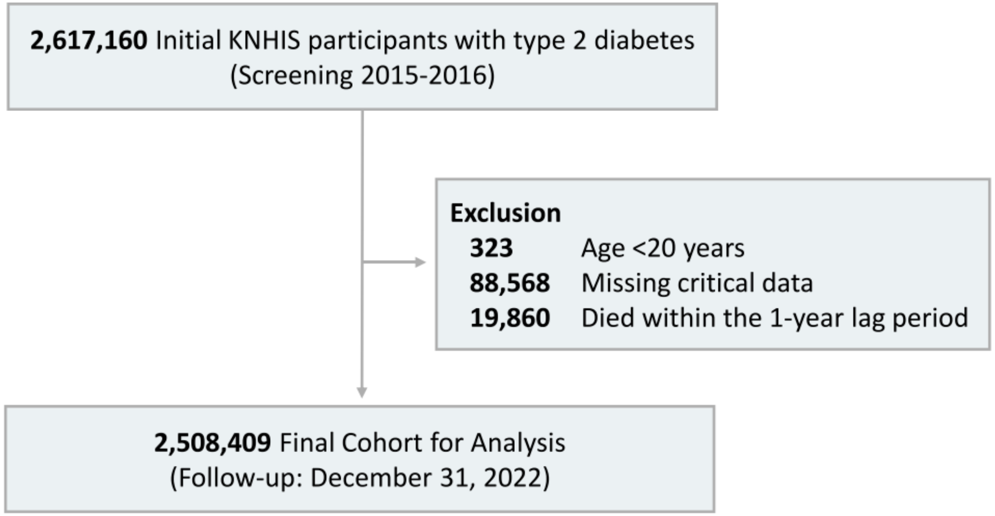

KNHIS, Korean National Health Insurance Service.

Supplementary Figure 2. Cumulative incidence of respiratory infection-related mortality by GLIM-based malnutrition staging in individuals with type 2 diabetes

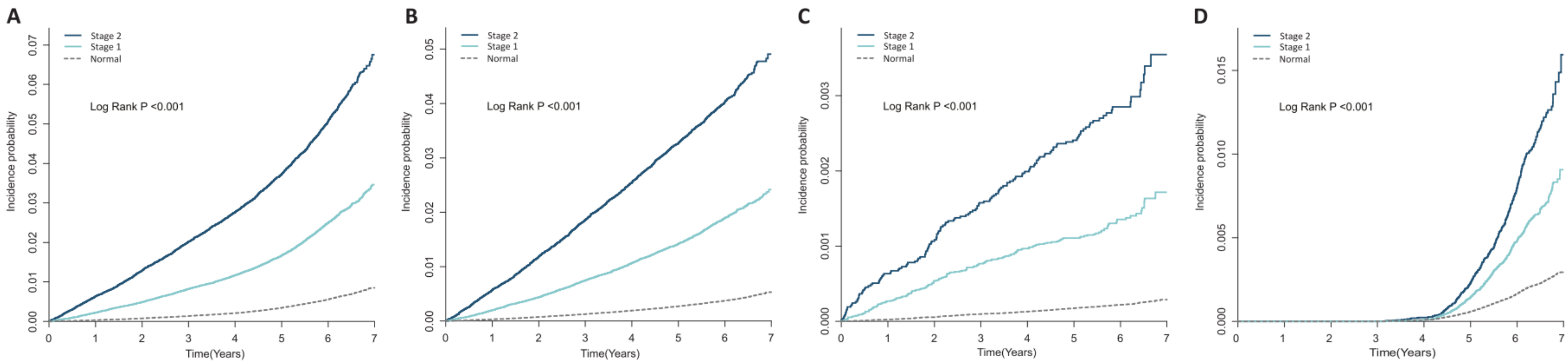

Kaplan–Meier survival curves showing cumulative incidence of (A) total respiratory infection-related mortality, (B) influenza and pneumonia mortality, (C) tuberculosis mortality, and (D) COVID-19 mortality according to the Global Leadership Initiative on Malnutrition (GLIM) phenotypic BMI thresholds. Participants were classified as having normal nutritional status, stage 1 malnutrition (BMI <20 kg/m^2 for age <70 years or <22 kg/m^2 for age ≥70 years), or stage 2 malnutrition (BMI <18.5 kg/m^2 for age <70 years or <20 kg/m^2 for age ≥70 years). Log-rank tests were used to compare cumulative incidence across groups (all P <0.001). A clear graded increase in cumulative incidence was observed with worsening malnutrition stage for all respiratory infection outcomes.

Table S1. Baseline characteristics of the participants stratified by underweight status

Variable	Total (kg/m²)	BMI Group								*P* for trend
		Severe Underweight	Moderate Underweight	Mild Underweight	Normal weight	Overweight	Class I Obesity	Class II Obesity	Class III Obesity	
		<16	16–16.9	17–18.4	18.5–22.9	23–24.9	25–29.9	30–34.9	≥35	
n	2,508,409	2,571	5,445	26,157	586,424	606,290	1,045,492	202,899	33,131	—
BMI (kg/m²)	25.3 ± 3.5	15.1 ± 0.8	16.5 ± 0.3	17.9 ± 0.4	21.4 ± 1.1	24.0 ± 0.6	27.0 ± 1.4	31.7 ± 1.3	37.6 ± 2.7	<0.001
Weight (kg)	67.4 ± 12.6	38.6 ± 5.1	42.6 ± 5.1	46.4 ± 5.4	56.3 ± 7.0	63.8 ± 7.3	72.1 ± 9.0	84.7 ± 11.1	100.6 ± 15.0	<0.001
Height (cm)	162.8 ± 9.4	159.5 ± 9.7	160.3 ± 9.4	160.9 ± 9.2	161.8 ± 9.0	162.8 ± 9.1	163.3 ± 9.4	163.3 ± 10.2	163.3 ± 11.1	<0.001
Waist circumference (cm)	86.2 ± 9.0	66.0 ± 7.5	67.7 ± 6.4	70.1 ± 5.9	78.0 ± 5.9	83.7 ± 5.4	89.6 ± 6.1	98.5 ± 6.9	108.8 ± 8.8	<0.001
Central obesity (n, %)	1,032,090 (41.2)	41 (1.6)	54 (1.0)	254 (1.0)	27,007 (4.6)	123,410(20.3)	652,986 (62.5)	195,516 (96.4)	32,822 (99.1)	<0.001
Age (years)	59.6 ± 12.0	65.4 ± 15.3	63.4 ± 15.0	62.1 ± 14.2	61.6 ± 11.8	60.9 ± 11.3	59.0 ± 11.6	54.5 ± 12.8	48.8 ± 13.5	<0.001
≥65 years (n, %)	864,687 (34.5)	1,372 (53.4)	2,614 (48.0)	11,536 (44.1)	235,646 (40.2)	226,074(37.3)	336,612 (32.2)	46,186 (22.8)	4,647 (14.0)	<0.001
Male (n, %)	1,510,820 (60.2)	1,274 (49.6)	2,855 (52.4)	13,993 (53.5)	334,035 (57.0)	379,254(62.6)	653,919 (62.6)	110,239 (54.3)	15,251 (46.0)	<0.001
≥3 oral antidiabetic drugs (n, %)	563,143 (22.5)	455 (17.7)	1,115 (20.5)	5,570 (21.3)	134,223 (22.9)	136,165(22.5)	231,958 (22.2)	46,230 (22.8)	7,427 (22.4)	0.0001
Insulin use (n, %)	205,191 (8.2)	371 (14.4)	818 (15.0)	3,607 (13.8)	57,895 (9.9)	48,804 (8.0)	77,041 (7.4)	14,380 (7.1)	2,275 (6.9)	<0.001
Hypertension (n, %)	1,477,384 (58.9)	1,038 (40.4)	2,220 (40.8)	10,490 (40.1)	286,328 (48.8)	342,197(56.4)	666,914 (63.8)	143,483 (70.7)	24,714 (74.6)	<0.001
Dyslipidemia (n, %)	1,436,734 (57.3)	842 (32.8)	1,825 (33.5)	9,776 (37.4)	303,701 (51.8)	347,000(57.2)	628,551 (60.1)	125,626 (61.9)	19,413 (58.6)	<0.001
Chronic kidney disease (n, %)	274,356 (10.9)	418 (16.3)	795 (14.6)	3,338 (12.8)	66,929 (11.4)	67,683 (11.2)	113,469 (10.9)	19,256 (9.5)	2,468 (7.5)	<0.001
History of CVD (n, %)	269,723 (10.8)	459 (17.9)	806 (14.8)	3,352 (12.8)	66,698 (11.4)	67,086 (11.1)	110,628 (10.6)	18,336 (9.0)	2,358 (7.1)	<0.001
Chronic heart failure (n, %)	148,103 (5.9)	222 (8.6)	433 (8.0)	1,686 (6.4)	32,921 (5.6)	34,186 (5.6)	62,697 (6.0)	13,567 (6.7)	2,391 (7.2)	<0.001
COPD (n, %)	171,383 (6.8)	485 (18.9)	887 (16.3)	3,124 (11.9)	45,673 (7.8)	41,547 (6.9)	66,440 (6.4)	11,580 (5.7)	1,647 (5.0)	<0.001
Chronic respiratory failure (n, %)	217 (0.0)	6 (0.2)	3 (0.1)	11 (0.0)	67 (0.0)	47 (0.0)	62 (0.0)	18 (0.0)	3 (0.0)	<0.001
Active cancer (n, %)*	111,837 (4.5)	192 (7.5)	461 (8.5)	1,923 (7.4)	31,606 (5.4)	27,528 (4.5)	41,940 (4.0)	7,150 (3.5)	1,037 (3.1)	<0.001
Active lung cancer (n, %)*	6,010 (0.2)	13 (0.5)	25 (0.5)	106 (0.4)	1,776 (0.3)	1,607 (0.3)	2,159 (0.2)	294 (0.1)	30 (0.1)	<0.001

Data are presented as mean ± standard deviation (SD) for continuous variables or n (%) for categorical variables. *Active cancer and active lung cancer were defined as those diagnosed within 5 years prior to study enrollment. BMI, body mass index; CVD, cardiovascular disease; COPD, chronic obstructive pulmonary disease.

Table S2. Baseline characteristics of participants aged ≥65 years stratified by underweight status

Variable	Total (kg/m²)	BMI group				*P* for trend
		Severe Underweight (<16)	Moderate Underweight (16–16.9)	Mild Underweight (17–18.4)	Non-underweight (≥18.5)	
n	864,687	1,372	2,614	11,536	849,165	
BMI (kg/m2)	24.7 ± 3.2	15.1 ± 0.8	16.5 ± 0.3	17.9 ± 0.4	24.9 ± 3.1	<0.001
Weight (kg)	62.2 ± 10.2	37.2 ± 4.9	41.0 ± 4.9	44.7 ± 5.3	62.6 ± 10.0	<0.001
Height (cm)	158.4 ± 8.9	156.8 ± 9.4	157.3 ± 9.3	157.9 ± 9.2	158.4 ± 8.9	<0.001
Waist circumference	86.0 ± 8.6	67.4 ± 7.9	68.8 ± 6.9	71.2 ± 6.2	86.2 ± 8.3	<0.001
Central obesity	376,870 (43.6)	36 (2.6)	42 (1.6)	195 (1.7)	376,597 (44.4)	<0.001
Age (years)	72.5 ± 5.4	77.2 ± 7.3	76.0 ± 7.0	74.9 ± 6.5	72.4 ± 5.3	<0.001
Male (n, %)	422,947 (48.9)	578 (42.1)	1,246 (47.7)	5,804 (50.3)	415,319 (48.9)	0.047
Systolic blood pressure (mmHg)	130.7 ± 15.4	122.0 ± 16.8	123.2 ± 17.2	125.1 ± 16.7	130.8 ± 15.3	<0.001
Diastolic blood pressure (mmHg)	76.3 ± 9.7	73.3 ± 10.2	73.5 ± 10.5	74.0 ± 10.3	76.4 ± 9.7	<0.001
Fasting glucose (mg/dL)	134.6 ± 39.6	138.7 ± 57.9	136.0 ± 52.4	136.7 ± 50.3	134.6 ± 39.3	<0.001
Total cholesterol (mg/dL)	175.2 ± 40.3	174.8 ± 40.5	173.0 ± 39.5	173.7 ± 39.7	175.2 ± 40.3	<0.001
Triglyceride* (mg/dL)	123.5 (123.4, 123.6)	93.8 (91.6, 96.0)	93.0 (91.3, 94.7)	95.2 (94.3, 96.1)	124.1 (124.0, 124.2)	<0.001
HDL-cholesterol (mg/dL)	50.5 ± 14.1	57.2 ± 17.1	57.0 ± 23.1	56.3 ± 16.2	50.4 ± 14.0	<0.001
LDL-cholesterol (mg/dL)	96.8 ± 35.8	96.7 ± 35.1	95.3 ± 34.6	95.6 ± 34.9	96.9 ± 35.8	0.001
AST (IU/L)	27.6 ± 18.1	27.1 ± 22.9	28.2 ± 24.0	27.4 ± 25.8	27.6 ± 18.0	0.889
ALT (IU/L)	25.4 ± 19.2	18.7 ± 14.9	19.9 ± 18.5	20.2 ± 19.3	25.5 ± 19.2	<0.001
eGFR (mL/min/1.73m²)	72.6 ± 18.1	73.3 ± 18.6	73.3 ± 19.2	73.9 ± 18.8	72.6 ± 18.1	<0.001
Social history						
Smoking (n, %)						
Never smoker	604,285 (69.9)	979 (71.4)	1,774 (67.9)	7,758 (67.2)	593,774 (69.9)	-
Ex-smoker	180,966 (20.9)	175 (12.8)	420 (16.1)	2,011 (17.4)	178,360 (21.0)	
Current smoker	79,436 (9.2)	218 (15.9)	420 (16.1)	1,767 (15.3)	77,031 (9.1)	

Alcohol (n, %)						
None	662,806 (76.7)	1,170 (85.3)	2,184 (83.5)	9,336 (80.9)	650,116 (76.6)	-
Mild	168,684 (19.5)	156 (11.4)	346 (13.2)	1,749 (15.2)	166,433 (19.6)	
Heavy	33,197 (3.8)	46 (3.4)	84 (3.2)	451 (3.9)	32,616 (3.8)	
Regular exercise (n, %)	190,331 (22.0)	117 (8.5)	333 (12.7)	1,912 (16.6)	187,969 (22.1)	<0.001
Low income (n, %)	169,996 (19.7)	278 (20.3)	530 (20.3)	2,331 (20.2)	166,857 (19.6)	0.101
Medical history						
Diabetes duration (years)						
New onset	141,467 (16.4)	399 (29.1)	642 (24.6)	2,472 (21.4)	137,954 (16.2)	-
< 5 years	178,767 (20.7)	232 (16.9)	472 (18.1)	1,997 (17.3)	176,066 (20.7)	
< 10 years	206,197 (23.9)	278 (20.3)	497 (19.0)	2,281 (19.8)	203,141 (23.9)	
≥ 10 years	338,256 (39.1)	463 (33.8)	1,003 (38.4)	4,786 (41.5)	332,004 (39.1)	
≥3 oral antidiabetic drugs	216,365 (25.0)	220 (16.0)	521 (19.9)	2,570 (22.3)	213,054 (25.1)	<0.001
Insulin use (n, %)	89,757 (10.4)	150 (10.9)	329 (12.6)	1,465 (12.7)	87,813 (10.3)	<0.001
Hypertension (n, %)	646,269 (74.7)	727 (53.0)	1,433 (54.8)	6,438 (55.8)	637,671 (75.1)	<0.001
Dyslipidemia (n, %)	536,288 (62.0)	470 (34.3)	995 (38.1)	4,912 (42.6)	529,911 (62.4)	<0.001
Chronic kidney disease (n, %)	197,590 (22.9)	330 (24.1)	619 (23.7)	2,515 (21.8)	194,126 (22.9)	0.714
History of CVD	165,140 (19.1)	347 (25.3)	609 (23.3)	2,391 (20.7)	161,793 (19.1)	<0.001
Chronic heart failure (n, %)	89,282 (10.3)	150 (10.9)	315 (12.1)	1,212 (10.5)	87,605 (10.3)	0.015
COPD (n, %)	109,062 (12.6)	356 (25.9)	625 (23.9)	2,202 (19.1)	105,879 (12.5)	<0.001
Chronic respiratory failure (n, %)	131 (0.0)	1 (0.1)	1 (0.0)	4 (0.0)	125 (0.0)	0.010
Active cancer[†]	60,392 (7.0)	135 (9.8)	311 (11.9)	1,203 (10.4)	58,743 (6.9)	<0.001
Active lung cancer[†]	4,086 (0.5)	10 (0.7)	19 (0.7)	82 (0.7)	3,975 (0.5)	<0.001

Data are presented as mean ± standard deviation (SD) for continuous variables or n (%) for categorical variables. *Triglycerides are presented as geometric mean (95% confidence interval) after log transformation due to right-skewed distribution. [†]Active cancer and active lung cancer were defined as those diagnosed within 5 years prior to study enrollment. BMI, body mass index; CVD, cardiovascular disease; COPD, chronic obstructive pulmonary disease.

Table S3. Risk of mortality from respiratory diseases across the BMI spectrum (reference: BMI 25.0–29.9 kg/m^2)

Outcome	BMI (kg/m²)	N	Event	Duration	IR per 1,000	Model 1	Model 2	Model 3
All Deaths due to	<16	2,571	196	11,680	16.78	22.71 (19.69–26.20)	10.13 (8.77–11.69)	10.32 (8.93–11.92)
Respiratory Diseases	16–16.9	5,445	271	26,933	10.06	13.23 (11.71–14.95)	6.89 (6.10–7.80)	6.77 (5.98–7.66)
	17–18.4	26,157	804	137,653	5.84	7.51 (6.97–8.09)	4.47 (4.15–4.82)	4.41 (4.09–4.76)
	18.5–22.9	586,424	7,065	3,323,037	2.13	2.66 (2.57–2.76)	1.96 (1.89–2.04)	1.97 (1.90–2.05)
	23–24.9	606,290	4,066	3,499,288	1.16	1.45 (1.39–1.51)	1.19 (1.14–1.24)	1.21 (1.16–1.26)
	25–29.9	1,045,492	4,859	6,064,899	0.80	1.00 (ref.)	1.00 (ref.)	1.00 (ref.)
	30–34.9	202,899	669	1,176,468	0.57	0.72 (0.66–0.78)	1.16 (1.07–1.25)	1.09 (1.00–1.18)
	≥35	33,131	94	191,099	0.49	0.62 (0.51–0.77)	1.95 (1.59–2.39)	1.69 (1.38–2.07)
	P value					<0.001	<0.001	<0.001
	P for trend					<0.001	<0.001	<0.001
Influenza/Pneumonia	<16	2,571	158	11,680	13.53	27.51 (23.44–32.27)	11.34 (9.66–13.32)	11.24 (9.56–13.21)
	16–16.9	5,445	227	26,933	8.43	16.85 (14.73–19.28)	8.19 (7.16–9.38)	7.81 (6.82–8.95)
	17–18.4	26,157	627	137,653	4.55	8.98 (8.24–9.79)	5.08 (4.66–5.53)	4.89 (4.48–5.34)
	18.5–22.9	586,424	5,307	3,323,037	1.60	3.10 (2.97–3.24)	2.23 (2.13–2.33)	2.22 (2.12–2.32)
	23–24.9	606,290	2,815	3,499,288	0.80	1.56 (1.48–1.64)	1.27 (1.20–1.33)	1.28 (1.22–1.35)
	25–29.9	1,045,492	3,130	6,064,899	0.52	1.00 (ref.)	1.00 (ref.)	1.00 (ref.)
	30–34.9	202,899	387	1,176,468	0.33	0.64 (0.58–0.71)	1.06 (0.95–1.18)	1.00 (0.90–1.11)
	≥35	33,131	52	191,099	0.27	0.53 (0.41–0.70)	1.75 (1.33–2.30)	1.52 (1.15–2.00)
	P value					<0.001	<0.001	<0.001
	P for trend					<0.001	<0.001	<0.001
Tuberculosis	<16	2,571	15	11,680	1.28	45.79 (27.01–77.62)	20.68 (12.14–35.21)	17.85 (10.43–30.54)
	16–16.9	5,445	13	26,933	0.48	17.15 (9.76–30.14)	8.93 (5.07–15.73)	7.73 (4.37–13.67)
	17–18.4	26,157	57	137,653	0.41	14.65 (10.86–19.77)	8.71 (6.44–11.79)	7.93 (5.83–10.80)
	18.5–22.9	586,424	363	3,323,037	0.11	3.85 (3.21–4.62)	2.83 (2.36–3.39)	2.82 (2.34–3.39)
	23–24.9	606,290	173	3,499,288	0.05	1.74 (1.41–2.15)	1.43 (1.15–1.76)	1.45 (1.18–1.80)
	25–29.9	1,045,492	172	6,064,899	0.03	1.00 (ref.)	1.00 (ref.)	1.00 (ref.)
	30–34.9	202,899	13	1,176,468	0.01	0.39 (0.22–0.69)	0.65 (0.37–1.13)	0.61 (0.34–1.06)
	≥35	33,131	2	191,099	0.01	0.37 (0.09–1.50)	1.20 (0.30–4.85)	1.04 (0.26–4.21)
	P value					<0.001	<0.001	<0.001
	P for trend					<0.001	<0.001	<0.001
COVID-19	<16	2,571	23	11,680	1.97	9.31 (6.17–14.05)	5.25 (3.48–7.92)	5.88 (3.89–8.89)
	16–16.9	5,445	31	26,933	1.15	5.06 (3.55–7.22)	3.22 (2.25–4.59)	3.52 (2.46–5.03)
	17–18.4	26,157	120	137,653	0.87	3.63 (3.02–4.38)	2.50 (2.08–3.02)	2.67 (2.21–3.22)
	18.5–22.9	586,424	1,395	3,323,037	0.42	1.65 (1.53–1.77)	1.30 (1.21–1.39)	1.33 (1.24–1.44)
	23–24.9	606,290	1,078	3,499,288	0.31	1.20 (1.11–1.29)	1.02 (0.94–1.10)	1.04 (0.96–1.13)
	25–29.9	1,045,492	1,557	6,064,899	0.26	1.00 (ref.)	1.00 (ref.)	1.00 (ref.)
	30–34.9	202,899	269	1,176,468	0.23	0.91 (0.80–1.03)	1.36 (1.20–1.55)	1.28 (1.13–1.46)
	≥35	33,131	40	191,099	0.21	0.85 (0.62–1.16)	2.27 (1.66–3.11)	1.97 (1.44–2.70)
	P value					<0.001	<0.001	<0.001
	P for trend					<0.001	<0.001	<0.001

Model 1: Unadjusted
Model 2: Adjusted for age and sex
Model 3: Adjusted for age, sex, income, smoking status, alcohol intake, physical activities, the presence of hypertension, dyslipidemia, chronic kidney disease, chronic heart failure, chronic obstructive pulmonary disease, chronic respiratory failure, active cancer diagnosed within five years, fasting glucose, use of three or more oral antidiabetic medication or insulin, duration of diabetes

Table S4. Risk of mortality from respiratory diseases across the BMI spectrum (reference: BMI 18.5–22.9 kg/m²)

Outcome	BMI (kg/m²)	N	Event	Duration	IR per 1,000	Model 1	Model 2	Model 3
All Deaths due to	<16	2,571	196	11,680	16.78	8.53 (7.40–9.83)	5.16 (4.48–5.95)	5.24 (4.54–6.04)
Respiratory Diseases	16–16.9	5,445	271	26,933	10.06	4.97 (4.40–5.61)	3.51 (3.11–3.97)	3.43 (3.04–3.88)
	17–18.4	26,157	804	137,653	5.84	2.82 (2.62–3.03)	2.28 (2.12–2.45)	2.24 (2.08–2.41)
	18.5–22.9	586,424	7,065	3,323,037	2.13	1.00 (ref.)	1.00 (ref.)	1.00 (ref.)
	23–24.9	606,290	4,066	3,499,288	1.16	0.54 (0.52–0.57)	0.61 (0.59–0.63)	0.61 (0.59–0.64)
	25–29.9	1,045,492	4,859	6,064,899	0.80	0.38 (0.36–0.39)	0.51 (0.49–0.53)	0.51 (0.49–0.53)
	30–34.9	202,899	669	1,176,468	0.57	0.27 (0.25–0.29)	0.59 (0.54–0.64)	0.55 (0.51–0.60)
	≥35	33,131	94	191,099	0.49	0.23 (0.19–0.29)	0.99 (0.81–1.22)	0.86 (0.70–1.05)
	P value					<0.001	<0.001	<0.001
	P for trend					<0.001	<0.001	<0.001
Influenza/Pneumonia	<16	2,571	158	11,680	13.53	8.87 (7.57–10.39)	5.09 (4.34–5.97)	5.06 (4.32–5.94)
	16–16.9	5,445	227	26,933	8.43	5.43 (4.76–6.21)	3.68 (3.22–4.20)	3.52 (3.08–4.02)
	17–18.4	26,157	627	137,653	4.55	2.90 (2.67–3.15)	2.28 (2.10–2.48)	2.20 (2.03–2.39)
	18.5–22.9	586,424	5,307	3,323,037	1.60	1.00 (ref.)	1.00 (ref.)	1.00 (ref.)
	23–24.9	606,290	2,815	3,499,288	0.80	0.50 (0.48–0.53)	0.57 (0.54–0.60)	0.58 (0.55–0.61)
	25–29.9	1,045,492	3,130	6,064,899	0.52	0.32 (0.31–0.34)	0.45 (0.43–0.47)	0.45 (0.43–0.47)
	30–34.9	202,899	387	1,176,468	0.33	0.21 (0.19–0.23)	0.48 (0.43–0.53)	0.45 (0.41–0.50)
	≥35	33,131	52	191,099	0.27	0.17 (0.13–0.23)	0.79 (0.60–1.03)	0.68 (0.52–0.90)
	P value					<0.001	<0.001	<0.001
	P for trend					<0.001	<0.001	<0.001
Tuberculosis	<16	2,571	15	11,680	1.28	11.90 (7.10–19.94)	7.32 (4.35–12.30)	6.34 (3.76–10.68)
	16–16.9	5,445	13	26,933	0.48	4.46 (2.56–7.75)	3.16 (1.81–5.50)	2.74 (1.57–4.79)
	17–18.4	26,157	57	137,653	0.41	3.81 (2.88–5.03)	3.08 (2.33–4.08)	2.82 (2.13–3.74)
	18.5–22.9	586,424	363	3,323,037	0.11	1.00 (ref.)	1.00 (ref.)	1.00 (ref.)
	23–24.9	606,290	173	3,499,288	0.05	0.45 (0.38–0.54)	0.50 (0.42–0.61)	0.52 (0.43–0.62)
	25–29.9	1,045,492	172	6,064,899	0.03	0.26 (0.22–0.31)	0.35 (0.30–0.43)	0.36 (0.30–0.43)
	30–34.9	202,899	13	1,176,468	0.01	0.10 (0.06–0.18)	0.23 (0.13–0.40)	0.21 (0.12–0.38)
	≥35	33,131	2	191,099	0.01	0.10 (0.02–0.39)	0.43 (0.11–1.71)	0.37 (0.09–1.49)
	P value					<0.001	<0.001	<0.001
	P for trend					<0.001	<0.001	<0.001
COVID-19	<16	2,571	23	11,680	1.97	5.67 (3.76–8.55)	4.05 (2.68–6.11)	4.41 (2.92–6.67)
	16–16.9	5,445	31	26,933	1.15	3.07 (2.15–4.38)	2.48 (1.74–3.54)	2.64 (1.85–3.77)
	17–18.4	26,157	120	137,653	0.87	2.21 (1.83–2.66)	1.93 (1.60–2.33)	2.00 (1.66–2.41)
	18.5–22.9	586,424	1,395	3,323,037	0.42	1.00 (ref.)	1.00 (ref.)	1.00 (ref.)
	23–24.9	606,290	1,078	3,499,288	0.31	0.73 (0.67–0.79)	0.79 (0.73–0.85)	0.78 (0.72–0.85)
	25–29.9	1,045,492	1,557	6,064,899	0.26	0.61 (0.56–0.65)	0.77 (0.72–0.83)	0.75 (0.70–0.81)
	30–34.9	202,899	269	1,176,468	0.23	0.55 (0.48–0.63)	1.05 (0.92–1.20)	0.96 (0.84–1.10)
	≥35	33,131	40	191,099	0.21	0.52 (0.38–0.71)	1.75 (1.28–2.40)	1.48 (1.08–2.03)
	P value					<0.001	<0.001	<0.001
	P for trend					<0.001	<0.001	<0.001

Model 1: Unadjusted
Model 2: Adjusted for age and sex
Model 3: Adjusted for age, sex, income, smoking status, alcohol intake, physical activities, the presence of hypertension, dyslipidemia, chronic kidney disease, chronic heart failure, chronic obstructive pulmonary disease, chronic respiratory failure, active cancer diagnosed within five years, fasting glucose, use of three or more oral antidiabetic medication or insulin, duration of diabetes

Table S5. Subgroup analysis for the risk of mortality from respiratory infection

Subgroup	BMI Group	N	Events	Duration	IR per 1,000	Fully adjusted model	*P* for interaction
Age <65	<16	1,199	38	6,223	6.11	21.37 (15.50–29.46)	<0.001
	16-16.9	2,831	47	15,428	3.05	10.44 (7.82–13.94)	
	17-18.4	14,621	120	81,988	1.46	5.22 (4.34–6.27)	
	≥18.5	1,625,071	2,383	9,505,052	0.25	1 (ref.)	
Age ≥65	<16	1,372	158	5,457	28.96	6.22 (5.31–7.28)	
	16-16.9	2,614	224	11,505	19.47	4.27 (3.74–4.88)	
	17-18.4	11,536	684	55,666	12.29	2.93 (2.71–3.16)	
	≥18.5	849,165	14,370	4,749,739	3.03	1 (ref.)	
Sex, Male	<16	1,274	100	5,559	17.99	7.62 (6.25–9.28)	0.600
	16-16.9	2,855	156	13,625	11.45	4.82 (4.11–5.65)	
	17-18.4	13,993	521	71,705	7.27	3.26 (2.98–3.56)	
	≥18.5	1,492,698	10,905	8,562,478	1.27	1 (ref.)	
Sex, Female	<16	1,297	96	6,120	15.69	6.94 (5.66–8.49)	
	16-16.9	2,590	115	13,308	8.64	4.76 (3.95–5.72)	
	17-18.4	12,164	283	65,948	4.29	2.97 (2.63–3.35)	
	≥18.5	981,538	5,848	5,692,313	1.03	1 (ref.)	
DM Duration, New onset	<16	871	45	4,144	10.86	5.40 (4.02–7.26)	0.010
	16-16.9	1,767	62	9,021	6.87	4.14 (3.21–5.33)	
	17-18.4	8,122	184	43,758	4.21	3.27 (2.81–3.80)	
	≥18.5	747,459	2,503	4,320,986	0.58	1 (ref.)	
DM Duration, <5 yrs	<16	491	36	2,293	15.7	10.13 (7.29–14.08)	
	16-16.9	982	53	4,976	10.65	6.16 (4.69–8.09)	
	17-18.4	4,690	140	24,964	5.61	3.75 (3.16–4.44)	
	≥18.5	587,953	2,738	3,430,069	0.8	1 (ref.)	
DM Duration, <10 yrs	<16	496	45	2,178	20.66	8.04 (5.99–10.80)	
	16-16.9	1,012	52	4,948	10.51	5.19 (3.95–6.83)	
	17-18.4	4,919	154	25,814	5.97	3.35 (2.85–3.94)	
	≥18.5	516,093	3,686	2,988,266	1.23	1 (ref.)	
DM Duration, ≥10 yrs	<16	713	70	3,063	22.85	7.36 (5.82–9.32)	
	16-16.9	1,684	104	7,987	13.02	4.52 (3.73–5.49)	
	17-18.4	8,426	326	43,118	7.56	2.81 (2.52–3.15)	
	≥18.5	622,731	7,826	3,515,469	2.23	1 (ref.)	
Glucose ≥200	<16	401	26	1,876	13.86	6.26 (4.25–9.23)	0.160
	16-16.9	741	39	3,570	10.92	6.44 (4.69–8.86)	
	17-18.4	3,456	94	17,884	5.26	2.88 (2.34–3.55)	
	≥18.5	237,398	1,712	1,359,975	1.26	1 (ref.)	
Glucose <200	<16	2,170	170	9,803	17.34	7.34 (6.30–8.55)	
	16-16.9	4,704	232	23,363	9.93	4.58 (4.02–5.22)	
	17-18.4	22,701	710	119,769	5.93	3.16 (2.93–3.41)	
	≥18.5	2,236,838	15,041	12,894,816	1.17	1 (ref.)	

Smoking, Non-Ex	<16	1,861	164	8,330	19.69	7.42 (6.36–8.67)	0.929
	16-16.9	3,888	215	19,151	11.23	4.79 (4.18–5.49)	
	17-18.4	18,749	650	98,700	6.59	3.16 (2.92–3.43)	
	≥18.5	1,919,123	14,403	11,070,678	1.3	1 (ref.)	
Smoking, Current	<16	710	32	3,349	9.55	6.58 (4.64–9.33)	
	16-16.9	1,557	56	7,782	7.2	4.80 (3.68–6.25)	
	17-18.4	7,408	154	38,953	3.95	3.08 (2.62–3.63)	
	≥18.5	555,113	2,350	3,184,112	0.74	1 (ref.)	
COPD, No	<16	2,086	144	9,846	14.62	7.96 (6.75–9.38)	0.050
	16-16.9	4,558	200	23,118	8.65	5.08 (4.42–5.85)	
	17-18.4	23,033	598	123,195	4.85	3.25 (3.00–3.53)	
	≥18.5	2,307,349	13,385	13,335,320	1	1 (ref.)	
COPD, Yes	<16	485	52	1,833	28.37	5.85 (4.45–7.70)	
	16-16.9	887	71	3,815	18.61	4.10 (3.24–5.19)	
	17-18.4	3,124	206	14,458	14.25	2.87 (2.49–3.30)	
	≥18.5	166,887	3,368	919,471	3.66	1 (ref.)	
CKD, No	<16	2,153	161	10,060	16	8.99 (7.69–10.52)	<0.001
	16-16.9	4,650	201	23,722	8.47	5.49 (4.78–6.32)	
	17-18.4	22,819	564	122,720	4.6	3.31 (3.04–3.61)	
	≥18.5	2,204,431	10,593	12,791,245	0.83	1 (ref.)	
CKD, Yes	<16	418	35	1,620	21.61	3.88 (2.78–5.41)	
	16-16.9	795	70	3,211	21.8	3.51 (2.78–4.45)	
	17-18.4	3,338	240	14,933	16.07	2.84 (2.49–3.23)	
	≥18.5	269,805	6,160	1,463,545	4.21	1 (ref.)	
Previous CVD, No	<16	2,112	134	9,966	13.45	7.64 (6.44–9.07)	0.205
	16-16.9	4,639	192	23,631	8.12	5.03 (4.35–5.80)	
	17-18.4	22,805	557	122,362	4.55	3.13 (2.88–3.42)	
	≥18.5	2,209,130	11,600	12,787,350	0.91	1 (ref.)	
Previous CVD, Yes	<16	459	62	1,713	36.19	5.99 (4.66–7.71)	
	16-16.9	806	79	3,301	23.93	4.12 (3.30–5.15)	
	17-18.4	3,352	247	15,292	16.15	3.15 (2.77–3.58)	
	≥18.5	265,106	5,153	1,467,440	3.51	1 (ref.)	
Chronic heart failure, No	<16	2,349	173	10,866	15.92	7.48 (6.43–8.70)	0.285
	16-16.9	5,012	226	25,170	8.98	4.85 (4.25–5.53)	
	17-18.4	24,471	697	130,174	5.35	3.23 (2.99–3.49)	
	≥18.5	2,328,474	13,998	13,459,024	1.04	1 (ref.)	
Chronic heart failure, Yes	<16	222	23	813	28.28	6.05 (4.01–9.13)	
	16-16.9	433	45	1,763	25.52	4.54 (3.38–6.10)	
	17-18.4	1,686	107	7,479	14.31	2.70 (2.23–3.28)	
	≥18.5	145,762	2,755	795,767	3.46	1 (ref.)	
Any active cancer, No	<16	2,379	181	10,868	16.66	7.38 (6.37–8.56)	0.564
	16-16.9	4,984	235	24,875	9.45	4.66 (4.10–5.31)	

	17-18.4	24,234	715	128,332	5.57	3.15 (2.92–3.40)	
	≥18.5	2,364,975	15,393	13,655,121	1.13	1 (ref.)	
Any active cancer, Yes	<16	192	15	812	18.47	6.17 (3.71–10.27)	
	16-16.9	461	36	2,057	17.5	5.85 (4.20–8.15)	
	17-18.4	1,923	89	9,321	9.55	3.15 (2.54–3.90)	
	≥18.5	109,261	1,360	599,670	2.27	1 (ref.)	
Active lung cancer, No	<16	2,558	195	11,631	16.77	7.29 (6.32–8.40)	0.918
	16-16.9	5,420	268	26,828	9.99	4.78 (4.23–5.40)	
	17-18.4	26,051	797	137,232	5.81	3.15 (2.93–3.39)	
	≥18.5	2,468,370	16,638	14,226,122	1.17	1 (ref.)	
Active lung cancer, Yes	<16	13	1	48	20.67	5.31 (0.74–38.06)	
	16-16.9	25	3	105	28.7	6.84 (2.20–21.33)	
	17-18.4	106	7	421	16.63	3.00 (1.40–6.41)	
	≥18.5	5,866	115	28,668	4.01	1 (ref.)	

Hazard ratios (HRs) and 95% confidence intervals (CIs) were estimated using Cox proportional hazards models. The fully adjusted model included age, sex, income, smoking status, alcohol intake, physical activities, the presence of hypertension, dyslipidemia, chronic kidney disease, chronic heart failure, chronic obstructive pulmonary disease, chronic respiratory failure, active cancer diagnosed within five years, fasting glucose, use of three or more oral antidiabetic medication or insulin, duration of diabetes. For each subgroup analysis, the stratification variable was not included in the corresponding multivariable model. P values for interaction were calculated by including an interaction term between BMI group and the subgroup variable. BMI, body mass index; CI, confidence interval; CKD, chronic kidney disease; COPD, chronic obstructive pulmonary disease; CVD, cardiovascular disease; DM, diabetes mellitus; HR, hazard ratio; IR, incidence rate.

Table S6. Risk of mortality from respiratory diseases according to BMI categories among individuals aged ≥65 years with diabetes

Outcome	BMI Group	N	Event	Duration	IR per 1,000	Model 1	Model 2	Model 3
All Deaths due to Respiratory Diseases	<16	1,372	158	5,457	29.0	10.79 (9.23–12.62)	6.01 (5.14–7.04)	5.98 (5.11–7.01)
	16–16.9	2,614	224	11,505	19.5	7.00 (6.13–7.99)	4.33 (3.79–4.94)	4.15 (3.64–4.75)
	17–18.4	11,536	684	55,666	12.3	4.27 (3.96–4.61)	2.97 (2.75–3.21)	2.87 (2.65–3.10)
	≥18.5	849,165	14,370	4,749,739	3.0	1 (ref.)	1 (ref.)	1 (ref.)
	P value					<0.001	<0.001	<0.001
	P for trend					<0.001	<0.001	<0.001
Influenza / Pneumonia	<16	1,372	130	5,457	23.8	11.88 (9.99–14.13)	6.28 (5.27–7.47)	6.11 (5.13–7.28)
	16–16.9	2,614	187	11,505	16.3	7.94 (6.87–9.18)	4.68 (4.05–5.41)	4.38 (3.78–5.07)
	17–18.4	11,536	539	55,666	9.7	4.64 (4.25–5.06)	3.11 (2.85–3.40)	2.95 (2.70–3.22)
	≥18.5	849,165	10,235	4,749,739	2.2	1 (ref.)	1 (ref.)	1 (ref.)
	P value					<0.001	<0.001	<0.001
	P for trend					<0.001	<0.001	<0.001
Tuberculosis	<16	1,372	12	5,457	2.2	17.16 (9.69–30.38)	9.33 (5.24–16.61)	8.01 (4.49–14.32)
	16–16.9	2,614	9	11,505	0.8	6.08 (3.15–11.75)	3.65 (1.88–7.06)	3.10 (1.60–6.03)
	17–18.4	11,536	44	55,666	0.8	6.12 (4.51–8.31)	4.15 (3.05–5.65)	3.68 (2.69–5.03)
	≥18.5	849,165	618	4,749,739	0.1	1 (ref.)	1 (ref.)	1 (ref.)
	P value					<0.001	<0.001	<0.001
	P for trend					<0.001	<0.001	<0.001
COVID-19	<16	1,372	16	5,457	2.9	5.31 (3.25–8.67)	3.58 (2.19–5.86)	3.88 (2.37–6.35)
	16–16.9	2,614	28	11,505	2.4	4.03 (2.78–5.84)	2.93 (2.02–4.25)	3.11 (2.14–4.52)
	17–18.4	11,536	101	55,666	1.8	2.77 (2.27–3.37)	2.15 (1.76–2.62)	2.24 (1.83–2.73)
	≥18.5	849,165	3,517	4,749,739	0.7	1 (ref.)	1 (ref.)	1 (ref.)
	P value					<0.001	<0.001	<0.001
	P for trend					<0.001	<0.001	<0.001

Model 1: Unadjusted

Model 2: Adjusted for age and sex
Model 3: Adjusted for age, sex, income, smoking status, alcohol intake, physical activities, the presence of hypertension, dyslipidemia, chronic kidney disease, chronic heart failure, chronic obstructive pulmonary disease, chronic respiratory failure, active cancer diagnosed within five years, fasting glucose, use of three or more oral antidiabetic medication or insulin, duration of diabetes

Table S7. Sensitivity analysis excluding the 1-year lag period

Outcome	BMI Group	N	Event	Duration	IR per 1,000	Model 1	Model 2	Model 3
All Deaths due to Respiratory Diseases	<16	3,011	257	14,460	17.8	18.85 (16.67–21.33)	8.59 (7.59–9.72)	8.36 (7.38–9.46)
	16–16.9	5,864	321	32,579	9.9	10.04 (8.99–11.21)	5.47 (4.89–6.11)	5.12 (4.58–5.72)
	17–18.4	27,259	935	164,368	5.7	5.63 (5.27–6.01)	3.56 (3.33–3.81)	3.38 (3.16–3.61)
	≥18.5	2,492,135	17,564	16,738,710	1.0	1 (ref.)	1 (ref.)	1 (ref.)
	P value					<0.001	<0.001	<0.001
	P for trend					<0.001	<0.001	<0.001
Influenza / Pneumonia	<16	3,011	209	14,460	14.5	20.92 (18.25–23.98)	8.77 (7.64–10.07)	8.28 (7.21–9.51)
	16–16.9	5,864	269	32,579	8.3	11.63 (10.30–13.12)	5.91 (5.23–6.67)	5.36 (4.74–6.05)
	17–18.4	27,259	733	164,368	4.5	6.16 (5.71–6.63)	3.71 (3.44–4.00)	3.43 (3.18–3.70)
	≥18.5	2,492,135	12,427	16,738,710	0.7	1 (ref.)	1 (ref.)	1 (ref.)
	P value					<0.001	<0.001	<0.001
	P for trend					<0.001	<0.001	<0.001
Tuberculosis	<16	3,011	25	14,460	1.7	36.93 (24.80–55.00)	16.93 (11.31–25.33)	13.48 (9.00–20.26)
	16–16.9	5,864	21	32,579	0.6	13.68 (8.87–21.09)	7.42 (4.80–11.47)	5.97 (3.85–9.26)
	17–18.4	27,259	82	164,368	0.5	10.53 (8.39–13.21)	6.63 (5.27–8.34)	5.67 (4.49–7.17)
	≥18.5	2,492,135	798	16,738,710	0.0	1 (ref.)	1 (ref.)	1 (ref.)
	P value					<0.001	<0.001	<0.001
	P for trend					<0.001	<0.001	<0.001
COVID-19	<16	3,011	23	14,460	1.6	7.83 (5.20–11.79)	4.70 (3.12–7.08)	5.16 (3.42–7.79)
	16–16.9	5,864	31	32,579	1.0	4.26 (2.99–6.06)	2.89 (2.03–4.11)	3.10 (2.17–4.41)
	17–18.4	27,259	120	164,368	0.7	3.06 (2.55–3.66)	2.25 (1.88–2.70)	2.35 (1.96–2.83)
	≥18.5	2,492,135	4,339	16,738,710	0.3	1 (ref.)	1 (ref.)	1 (ref.)
	P value					<0.001	<0.001	<0.001
	P for trend					<0.001	<0.001	<0.001

Model 1: Unadjusted
Model 2: Adjusted for age and sex

Model 3: Adjusted for age, sex, income, smoking status, alcohol intake, physical activities, the presence of hypertension, dyslipidemia, chronic kidney disease, chronic heart failure, chronic obstructive pulmonary disease, chronic respiratory failure, active cancer diagnosed within five years, fasting glucose, use of three or more oral antidiabetic medication or insulin, duration of diabetes

Table S8. Sensitivity analysis with cardiovascular, diabetes, and cancer mortality as competing risks

Outcome	BMI Group	N	Event	Duration	IR per 1,000	Model 1	Model 2	Model 3
All Deaths due to Respiratory Diseases	<16	2,571	196	13,121	14.9	13.63 (11.82–15.72)	6.04 (5.17–7.06)	5.83 (4.99–6.82)
	16–16.9	5,445	271	29,375	9.2	8.28 (7.34–9.34)	4.20 (3.67–4.80)	3.93 (3.44–4.48)
	17–18.4	26,157	804	146,741	5.5	4.84 (4.51–5.19)	2.89 (2.69–3.12)	2.74 (2.54–2.95)
	≥18.5	2,474,236	16,753	14,548,043	1.2	1.00 (ref.)	1.00 (ref.)	1.00 (ref.)
	P value					<0.001	<0.001	<0.001
	P for trend					<0.001	<0.001	<0.001
Influenza / Pneumonia	<16	2,571	158	13,121	12.0	15.42 (13.16–18.07)	6.43 (5.41–7.65)	6.05 (5.09–7.18)
	16–16.9	5,445	227	29,375	7.7	9.81 (8.60–11.19)	4.69 (4.05–5.43)	4.26 (3.68–4.93)
	17–18.4	26,157	627	146,741	4.3	5.37 (4.96–5.82)	3.07 (2.82–3.34)	2.83 (2.60–3.09)
	≥18.5	2,474,236	11,691	14,548,043	0.8	1.00 (ref.)	1.00 (ref.)	1.00 (ref.)
	P value					<0.001	<0.001	<0.001
	P for trend					<0.001	<0.001	<0.001
Tuberculosis	<16	2,571	15	13,121	1.1	23.16 (13.89–38.62)	10.52 (6.21–17.83)	8.53 (5.01–14.52)
	16–16.9	5,445	13	29,375	0.4	8.96 (5.18–15.50)	4.61 (2.64–8.07)	3.76 (2.14–6.62)
	17–18.4	26,157	57	146,741	0.4	7.84 (5.99–10.26)	4.72 (3.57–6.24)	4.05 (3.06–5.37)
	≥18.5	2,474,236	723	14,548,043	0.0	1.00 (ref.)	1.00 (ref.)	1.00 (ref.)
	P value					<0.001	<0.001	<0.001
	P for trend					<0.001	<0.001	<0.001
COVID-19	<16	2,571	23	13,121	1.8	6.59 (4.37–9.92)	3.41 (2.25–5.17)	3.71 (2.44–5.62)
	16–16.9	5,445	31	29,375	1.1	3.81 (2.68–5.43)	2.26 (1.59–3.23)	2.37 (1.66–3.38)
	17–18.4	26,157	120	146,741	0.8	2.85 (2.38–3.42)	1.92 (1.60–2.31)	1.98 (1.65–2.38)
	≥18.5	2,474,236	4,339	14,548,043	0.3	1.00 (ref.)	1.00 (ref.)	1.00 (ref.)
	P value					<0.001	<0.001	<0.001
	P for trend					<0.001	<0.001	<0.001

Competing risks included cardiovascular disease mortality (ICD-10 codes I), diabetes-related mortality (ICD-10 codes E), and cancer mortality (ICD-10 codes C).
Model 1: Unadjusted

Model 2: Adjusted for age and sex
Model 3: Adjusted for age, sex, income, smoking status, alcohol intake, physical activities, the presence of hypertension, dyslipidemia, chronic kidney disease, chronic heart failure, chronic obstructive pulmonary disease, chronic respiratory failure, active cancer diagnosed within five years, fasting glucose, use of three or more oral antidiabetic medication or insulin, duration of diabetes

Table S9. Baseline characteristics of participants according to GLIM-defined malnutrition stage

	BMI Group			*P* value	*P* for trend
	Normal	Stage 1 Malnutrition	Stage 2 Malnutrition		
n	2,318,459	131,997	57,953		
BMI (kg/m²)	25.8 ± 3.3	20.4 ± 1.0	18.2 ± 1.2	<0.001	<0.001
Weight (kg)	68.8 ± 11.9	52.1 ± 5.9	46.3 ± 5.9	<0.001	<0.001
Height (cm)	163.1 ± 9.3	159.8 ± 9.2	159.3 ± 9.4	<0.001	<0.001
Waist circumference	87.1 ± 8.5	76.2 ± 6.3	71.5 ± 6.6	<0.001	<0.001
Central obesity	1,025,760 (44.2)	5,312 (4.0)	1,018 (1.8)	<0.001	<0.001
Age (years)	58.9 ± 11.7	67.4 ± 12.4	68.3 ± 13.5	<0.001	<0.001
≥65 years (n, %)	738,501 (31.9)	86,884 (65.8)	39,302 (67.8)	<0.001	<0.001
Male (n, %)	1,413,718 (61.0)	67,733 (51.3)	29,369 (50.7)	<0.001	<0.001
Systolic blood pressure (mmHg)	128.7 ± 14.9	125.9 ± 16.1	123.8 ± 16.7	<0.001	<0.001
Diastolic blood pressure (mmHg)	78.4 ± 9.9	74.8 ± 9.9	74.1 ± 10.3	<0.001	<0.001
Fasting glucose (mg/dL)	144.8 ± 45.1	141.0 ± 51.0	143.5 ± 58.2	<0.001	<0.001
Total cholesterol (mg/dL)	185.9 ± 43.9	177.0 ± 41.1	175.8 ± 40.7	<0.001	<0.001
Triglyceride* (mg/dL)	140.8 (140.7, 140.9)	107.0 (106.7, 107.3)	98.0 (97.6, 98.5)	<0.001	<0.001
HDL-cholesterol (mg/dL)	50.6 ± 14.5	54.5 ± 16.5	57.0 ± 17.4	<0.001	<0.001
LDL-cholesterol (mg/dL)	103.5 ± 38.6	97.9 ± 36.1	96.2 ± 35.7	<0.001	<0.001
AST (IU/L)	30.1 ± 21.6	27.3 ± 26.1	29.0 ± 33.1	<0.001	<0.001
ALT (IU/L)	32.6 ± 26.9	22.5 ± 21.8	21.9 ± 23.9	<0.001	<0.001
eGFR (mL/min/1.73m²)	84.5 ± 20.1	78.9 ± 20.9	79.0 ± 21.5	<0.001	<0.001
Social history					
Smoking (n, %)				<0.001	-
Never smoker	1,259,328 (54.3)	84,204 (63.8)	36,760 (63.4)		
Ex-smoker	531,738 (22.9)	22,721 (17.2)	8,870 (15.3)		
Current smoker	527,393 (22.8)	25,072 (19.0)	12,323 (21.3)		
Alcohol (n, %)				<0.001	-
None	1,314,133 (56.7)	94,001 (71.2)	42,722 (73.7)		
Mild	787,369 (34.0)	30,576 (23.2)	11,975 (20.7)		
Heavy	216,957 (9.4)	7,420 (5.6)	3,256 (5.6)		
Regular exercise (n, %)	506,369 (21.8)	27,621 (20.9)	9,963 (17.2)	<0.001	<0.001
Low income (n, %)	499,655 (21.6)	27,784 (21.1)	13,099 (22.6)	<0.001	0.019
Medical history					
Diabetes duration (years)				<0.001	-
New onset	714,429 (30.8)	28,977 (21.9)	14,813 (25.6)		
< 5 years	560,122 (24.2)	23,857 (18.1)	10,137 (17.5)		
< 10 years	484,051 (20.9)	27,253 (20.6)	11,216 (19.4)		
≥ 10 years	559,857 (24.1)	51,910 (39.3)	21,787 (37.6)		
≥3 oral antidiabetic drugs	518,648 (22.4)	31,470 (23.8)	13,025 (22.5)	<0.001	<0.001
Insulin use (n, %)	182,279 (7.9)	15,289 (11.6)	7,623 (13.2)	<0.001	<0.001

Glucocorticoid use (n, %)					
Hypertension (n, %)	1,376,950 (59.4)	71,654 (54.3)	28,780 (49.7)	<0.001	<0.001
Dyslipidemia (n, %)	1,346,496 (58.1)	65,892 (49.9)	24,346 (42.0)	<0.001	<0.001
Chronic kidney disease (n, %)	240,790 (10.4)	22,952 (17.4)	10,614 (18.3)	<0.001	<0.001
History of CVD	239,039 (10.3)	20,763 (15.7)	9,921 (17.1)	<0.001	<0.001
Chronic heart failure (n, %)	132,803 (5.7)	10,363 (7.8)	4,937 (8.5)	<0.001	<0.001
COPD (n, %)	148,096 (6.4)	14,830 (11.2)	8,457 (14.6)	<0.001	<0.001
Chronic respiratory failure (n, %)	158 (0.0)	31 (0.0)	28 (0.1)	<0.001	<0.001
Active cancer (n, %)	97,802 (4.2)	9,194 (7.0)	4,841 (8.3)	<0.001	<0.001
Active lung cancer (n, %)	5,155 (0.2)	558 (0.4)	297 (0.5)	<0.001	<0.001

Data are presented as mean ± standard deviation (SD) for continuous variables or n (%) for categorical variables. Triglyceride levels are presented as median (95% confidence interval). *P* values were calculated using analysis of variance or the Kruskal–Wallis test for continuous variables and the chi-square test for categorical variables. *P* for trend was assessed using linear regression for continuous variables and the Cochran–Armitage trend test for categorical variables. Malnutrition stages were defined according to the Global Leadership Initiative on Malnutrition (GLIM) criteria. BMI, body mass index; HDL, high-density lipoprotein; LDL, low-density lipoprotein; AST, aspartate aminotransferase; ALT, alanine aminotransferase; eGFR, estimated glomerular filtration rate; CVD, cardiovascular disease; COPD, chronic obstructive pulmonary disease.

Table S10. Risk of respiratory disease–related mortality according to GLIM-defined malnutrition stage in individuals with diabetes

Outcome	BMI Group	N	Event	Duration	IR per 1,000	Model 1	Model 2	Model 3
All Deaths due to	Stage 2	57,953	2,518	295,611	8.5	9.47 (9.08–9.89)	3.39 (3.24–3.54)	3.32 (3.18–3.47)
Respiratory Diseases	Stage 1	131,997	2,962	721,116	4.1	4.46 (4.29–4.64)	1.79 (1.71–1.86)	1.78 (1.71–1.86)
	Normal	2,318,459	12,544	13,414,330	0.9	1.00 (ref.)	1.00 (ref.)	1.00 (ref.)
	P value					<0.001	<0.001	<0.001
	P for trend					<0.001	<0.001	<0.001
Influenza/Pneumonia	Stage 2	57,953	2,021	295,611	6.8	11.14 (10.61–11.70)	3.80 (3.61–4.00)	3.65 (3.46–3.84)
	Stage 1	131,997	2,257	721,116	3.1	5.03 (4.80–5.27)	1.94 (1.85–2.04)	1.92 (1.83–2.02)
	Normal	2,318,459	8,425	13,414,330	0.6	1.00 (ref.)	1.00 (ref.)	1.00 (ref.)
	P value					<0.001	<0.001	<0.001
	P for trend					<0.001	<0.001	<0.001
Tuberculosis	Stage 2	57,953	146	295,611	0.5	13.39 (11.13–16.10)	5.01 (4.13–6.07)	4.58 (3.76–5.58)
	Stage 1	131,997	165	721,116	0.2	6.18 (5.18–7.37)	2.58 (2.15–3.09)	2.54 (2.12–3.05)
	Normal	2,318,459	497	13,414,330	0.0	1.00 (ref.)	1.00 (ref.)	1.00 (ref.)
	P value					<0.001	<0.001	<0.001
	P for trend					<0.001	<0.001	<0.001
COVID-19	Stage 2	57,953	351	295,611	1.2	6.59 (4.37–9.92)	3.41 (2.25–5.17)	3.71 (2.44–5.62)
	Stage 1	131,997	540	721,116	0.7	3.81 (2.68–5.43)	2.26 (1.59–3.23)	2.37 (1.66–3.38)
	Normal	2,318,459	3,622	13,414,330	0.3	1.00 (ref.)	1.00 (ref.)	1.00 (ref.)
	P value					<0.001	<0.001	<0.001
	P for trend					<0.001	<0.001	<0.001

Values are presented as hazard ratios with 95% confidence intervals unless otherwise indicated. Incidence rates (IRs) are expressed per 1,000 person-years. Malnutrition stages were defined according to the Global Leadership Initiative on Malnutrition (GLIM) criteria, with Stage 1 and Stage 2 corresponding to moderate and severe malnutrition, respectively. Normal nutritional status was used as the reference group.

Model 1: Unadjusted

Model 2: Adjusted for age and sex

Model 3: Adjusted for age, sex, income, smoking status, alcohol intake, physical activities, the presence of hypertension, dyslipidemia, chronic kidney disease, chronic heart failure, chronic obstructive pulmonary disease, chronic respiratory failure, active cancer diagnosed within five years, fasting glucose, use of three or more oral antidiabetic medication or insulin, duration of diabetes

Table S11. Risk of respiratory disease–related mortality according to GLIM-defined malnutrition stage among individuals aged ≥65 years with diabetes

Outcome	BMI Group	N	Event	Duration	IR per 1,000	Model 1	Model 2	Model 3
All Deaths due to	Stage 2	39,302	2,313	191,972	12	5.09 (4.87–5.33)	3.09 (2.95–3.24)	3.05 (2.91–3.19)
Respiratory Diseases	Stage 1	86,884	2,747	462,484	5.9	2.44 (2.34–2.54)	1.67 (1.60–1.75)	1.67 (1.60–1.75)
	Normal	738,501	10,376	4,167,910	2.5	1.00 (ref.)	1.00 (ref.)	1.00 (ref.)
	P value					<0.001	<0.001	<0.001
	P for trend					<0.001	<0.001	<0.001
Influenza/Pneumonia	Stage 2	39,302	1,865	191,972	9.7	5.87 (5.58–6.18)	3.45 (3.28–3.64)	3.36 (3.18–3.54)
	Stage 1	86,884	2,109	462,484	4.6	2.71 (2.58–2.84)	1.82 (1.73–1.91)	1.81 (1.72–1.90)
	Normal	738,501	7,117	4,167,910	1.7	1.00 (ref.)	1.00 (ref.)	1.00 (ref.)
	P value					<0.001	<0.001	<0.001
	P for trend					<0.001	<0.001	<0.001
Tuberculosis	Stage 2	39,302	126	191,972	0.7	6.69 (5.48–8.16)	4.04 (3.29–4.96)	3.75 (3.04–4.63)
	Stage 1	86,884	145	462,484	0.3	3.18 (2.63–3.84)	2.18 (1.80–2.64)	2.16 (1.78–2.62)
	Normal	738,501	412	4,167,910	0.1	1.00 (ref.)	1.00 (ref.)	1.00 (ref.)
	P value					<0.001	<0.001	<0.001
	P for trend					<0.001	<0.001	<0.001
COVID-19	Stage 2	39,302	322	191,972	1.7	2.77 (2.47–3.10)	1.86 (1.65–2.09)	1.93 (1.71–2.17)
	Stage 1	86,884	493	462,484	1.1	1.64 (1.49–1.81)	1.20 (1.09–1.32)	1.22 (1.11–1.35)
	Normal	738,501	2,847	4,167,910	0.7	1.00 (ref.)	1.00 (ref.)	1.00 (ref.)
	P value					<0.001	<0.001	<0.001
	P for trend					<0.001	<0.001	<0.001

Values are presented as hazard ratios with 95% confidence intervals unless otherwise indicated. Incidence rates (IRs) are expressed per 1,000 person-years. Malnutrition stages were defined according to the Global Leadership Initiative on Malnutrition (GLIM) criteria, with Stage 1 and Stage 2 corresponding to moderate and severe malnutrition, respectively. Normal nutritional status was used as the reference group.
Model 1: Unadjusted
Model 2: Adjusted for age and sex
Model 3: Adjusted for age, sex, income, smoking status, alcohol intake, physical activities, the presence of hypertension, dyslipidemia, chronic kidney disease, chronic heart failure, chronic obstructive pulmonary disease, chronic respiratory failure, active cancer diagnosed within five years, fasting glucose, use of three or more oral antidiabetic medication or insulin, duration of diabetes
